# Modification and optimization of the FECPAK^G2^ protocol for the detection and quantification of soil-transmitted helminth eggs in human stool

**DOI:** 10.1371/journal.pntd.0006655

**Published:** 2018-10-15

**Authors:** Mio Ayana, Johnny Vlaminck, Piet Cools, Shaali Ame, Marco Albonico, Daniel Dana, Jennifer Keiser, Helen Manly, Leonardo F. Matoso, Zeleke Mekonnen, Antonio Montresor, Rodrigo Correa-Oliveira, Laura Rinaldi, Somphou Sayasone, Stephen Sowersby, Lensa Tesfaye, Jozef Vercruysse, Greg Mirams, Bruno Levecke

**Affiliations:** 1 Institute of Health, Faculty of Health Science, School of Medical Laboratory Science, Jimma University, Jimma, Ethiopia; 2 Department of Virology, Parasitology and Immunology, Ghent University, Merelbeke, Belgium; 3 Public Health Laboratory-Ivo de Carneri, Chake Chake, United Republic of Tanzania; 4 Center for Tropical Diseases, Sacro Cuore Don Calabria Hospital, Negrar, Italy; 5 Department of Life Sciences and Systems Biology, University of Turin, Turin, Italy; 6 Swiss Tropical and Public Health Institute, Basel, Switzerland; 7 Techion Group Ltd, Dunedin, New Zealand; 8 Laboratory of Molecular and Cellular Immunology, Research Center René Rachou—FIOCRUZ, Belo Horizonte, Brazil; 9 Nursing school, Federal university of Minas Gerais, Belo Horizonte, Brazil; 10 World Health Organization, Geneva, Switzerland; 11 Department of Veterinary Medicine and Animal Production, University of Naples Federico II, Naples, Italy; 12 National Institute of Public Health, Ministry of Health, Vientiane, Lao People's Democratic Republic; 13 Department of Biochemistry, University of Otago, Otago, New Zealand; Australian National University, AUSTRALIA

## Abstract

**Background:**

Standard diagnosis of human soil-transmitted helminth (STH) infections is based on the microscopic detection of helminth eggs in stool and supports programmatic decision making in control programs. However, the current standard diagnostic techniques still show a number of limitations. Recently, the FECPAK^G2^ method was developed to detect helminth infections and asses drug efficacy in sheep or cattle. It includes a device that takes digital images of helminth eggs that have been concentrated into one microscopic field of view and stores these images online for future evaluation. The goal of this study was to introduce a standard operating procedure (SOP) for the detection and quantification of human STH eggs using the FECPAK^G2^ and to optimize 2 crucial steps of the protocol, namely the sedimentation step (aimed at separating sinking eggs from floating debris) and the accumulation step (aimed at concentrating the eggs by flotation).

**Methodology/Principal findings:**

A total of 55 stool samples from naturally infected children were used from 4 different geographical areas (Ethiopia, Laos, Tanzania and Brazil). The results showed that *Trichuris* eggs generally moved slower than eggs of the other two STH species during both sedimentation in water in the FECPAK^G2^ sedimenter as during accumulation in flotation solution in the FECPAK^G2^ cassettes. The highest number of eggs were present in the slurry of the sedimenter after overnight sedimentation (*Ascaris*: 95.7%, *Trichuris*: 89.8% and hookworm: 94.2% of the eggs). A minimum of 24 minutes were needed to ensure the accumulation of at least 80% of the eggs from all three STH species in the FECPAK^G2^ cassette (*Ascaris*: 96.1%; *Trichuris*: 88.2% and hookworm: 87.6%).

**Conclusions/Significance:**

This study introduces for the first time a SOP for the FECPAK^G2^ method. Different aspects of the method for diagnosing human STH infections were optimized. Our study forms the basis for a thorough and objective evaluation of the system as a diagnostic tool that could be implemented in STH control programs.

## Author summary

Today, infections with soil-transmitted helminths are diagnosed by looking for the eggs of these helminths in stool samples using different microscopic techniques. However, these standard diagnostic techniques still show a number of limitations. The FECPAK^G2^ method was recently developed for diagnosing worm infections in the veterinary field and could also be useful to diagnose human helminth infections. This method produces digital images of helminth eggs that have been concentrated into one microscopic field of view and stores these images online. This eliminates the need for skilled technicians and opens the possibility for standardized quality control and possibly even automated egg counting by egg recognition software. This study presents, for the first time, a standard operating FECPAK^G2^ procedure for the detection and quantification of human STH eggs. We optimized the sedimentation and accumulation steps in the FECPAK^G2^ procedure for the detection of human helminth eggs using stool samples from naturally infected children. This established protocol allows for a thorough and objective evaluation of the system as a diagnostic tool that shows potential for implementation in future STH control programs.

## Introduction

Soil-transmitted helminths (STH) are a group of intestinal parasitic worms that infect humans through contact with infectious stages present in the soil. The main STH species are the giant roundworm (*Ascaris lumbricoides*), the whipworm (*Trichuris trichiura*), and the two hookworms (*Necator americanus* and *Ancylostoma duodenale*) [1]. Today, infections with STHs still pose an important risk to public health [2], particularly in subtropical and tropical countries where conditions are optimal for both the development of, and contact with, the infectious stages (e.g. lack of safe drinking water, environmental sanitation and hygiene [3–5].

To reduce the morbidity caused by STHs in endemic countries, the World Health Organization (WHO) recommends preventive chemotherapy (PC) programs, during which anthelmintic drugs are periodically administered to at-risk populations (i.e., preschool- and school-age children and women of reproductive age). Fueled by the London Declaration on Neglected Tropical Diseases (NTDs), the global coverage of children in PC programs has increased from ~30% in 2011 to 63.6% in 2016 [6], with the ultimate goals of covering at least 75% of children at risk and eliminating soil-transmitted helminthiasis as a public health problem in all endemic countries by 2020 [7].

Throughout STH control programs, diagnostic tools are essential for evidence-based decision-making, supporting program managers to (i) determine the type of PC (individual-based deworming *vs*. mass drug administration (MDA); annual *vs*. biannual PC [8]), (ii) to measure progress and to determine when scaling-down of deworming is justified [8], and (iii) to detect any change in therapeutic efficacy that may arise through the evolution of anthelmintic resistance [9].

The current standard to assess both the prevalence and intensity of STH infections is the demonstration and quantification of STH eggs in stool using microscopy. A variety of diagnostic methods have been deployed for processing human stool of which Kato-Katz thick smear remains the sole method recommended by WHO [8, 9]. However, there is a pressing need for improved diagnostic tools. In order to objectively evaluate existing technologies and prioritize product development for STH programmatic use, a group of global STH diagnostics experts recently provided a comprehensive framework that links program decision points to use-cases and their corresponding target product profiles (TPPs) [10]. For coproscopy, three opportunities for improved diagnosis were identified, including (i) integration of quality control / quality assurance for sample processing and egg counting, (ii) increased throughput, and (iii) internet connectivity to make test results accessible for remote interpretation.

A diagnostic platform that could potentially meet these 3 technical requirements is the FECPAK^G2^ platform [11]. The FECPAK^G2^ is a complete remote-location diagnostic tool that was recently launched for farmers and their veterinarians to assess both helminth infections and drug efficacy in sheep or cattle [12]. The platform includes a device (the MICRO-I) that is able to take digital images of helminth eggs that have been concentrated into one microscopic field of view [13]. Images are stored by the associated software and can be uploaded to a remote server when an internet connection is available. Later, a web-based lab technician can count the eggs in the images, after which the results are returned to the user by e-mail. The FECPAK^G2^ platform thus eliminates the need for trained microscopists in STH endemic settings with limited resources. The connectivity allows easy access for quality control of egg counts and the production of standardized analysis and reports. Maybe most importantly, it opens the door to automated egg counting by egg recognition software [14]. Such feature could further increase throughput, reduce personnel costs and variation in egg counts between technicians or laboratories.

Given the obvious similarities between the morphology of veterinary and human helminth eggs, FECPAK^G2^ also holds promise as a complete remote-location tool in STH control programs. However, a number of essential steps in the FECPAK^G2^ protocol established for veterinary practice still require additional refinement to detect human STH eggs. The main goal of this study was to develop a FECPAK^G2^ SOP for the detection and quantification of human STH eggs which includes the optimization of two crucial steps of the protocol (the sedimentation and accumulation step).

## Methods

### Ethics statement

The optimization of the FECPAK^G2^ protocol was part of a larger study aimed at evaluating three stool-based diagnostic methods (www.starworms.org). This study protocol was approved by the Institutional Review Board of Gent University (Ref. No B670201627755) and at each study site (Ethiopia: RPGC/547/2016, Laos: 018/NECHR; Zanzibar, Tanzania: ZAMREC/0002/February/2015 and Brazil: 2.037.205). Healthy children whose parents or legal guardians signed an informed consent and those who volunteered to provide a sufficient amount of stool were included. Students with active diarrhea, acute medical conditions or who had received anthelmintic treatment within 90 days prior to the collection date were excluded from participation. All children providing a stool sample were treated with a single oral dose of 400 mg albendazole (GlaxoSmithKline, batch number: 335726).

### Immediate changes in the FECPAK^G2^ Standard operating procedure

The FECPAK^G2^ was initially designed for the assessment of helminth eggs in feces from ruminants (sheep and cattle). Given the obvious differences in volume and consistency between feces from animals and humans, three immediate changes were introduced into the SOP before further optimizing the FECPAK^G2^ SOP for human stool (Table 1). First, the amount of human stool was fixed at 3 grams, whereas this varies from 2.4 to 6 grams for animals, depending on the species. Second, the human stool was homogenized using the Fill-FLOTAC device, which is part of the Mini-FLOTAC method [15], rather than homogenization in a zip lock plastic bag as is the case for animal feces. Using the Fill-FLOTAC device generally resulted in a much better homogenization of the human stool sample. Finally, given the difference in fecal matter consistency between ruminants and humans, a reduction in sieve mesh size was necessary. The outer and inner mesh size was reduced from 600 and 425 μm for animals to 425 and 250 μm for humans respectively. This resulted in significantly less small debris in the sample and thus clearer images of the FECPAK^G2^ wells.

**Table 1:** Comparison of FECPAK^G2^ standard operating procedures for analyzing animal and human stool.

Aspects of SOP	Animal stool	Human stool
**Stool quantity**	2.4–6 grams	3 grams
**Homogenizing stool**	In a zip lock plastic bag	In a Fill-FLOTAC device
**Sedimentation**	30 min in 210 ml water	≥1 hour in 210 ml water
**Volume of retained slurry**	15 ml	15 ml
**Total volume in filtration unit**	95–115 ml	95 ml
**Flotation solution**	Saturated saline (specific gravity of approximately 1.20)	
**Sieve mesh sizes**	600 and 425 μm	425 and 250 μm
**Volume of wells**	455 μl	455 μl
Accumulation time	≥6 min	≥24 min

### General FECPAK^G2^ SOP for analyzing human stool samples

**Fig 1:**
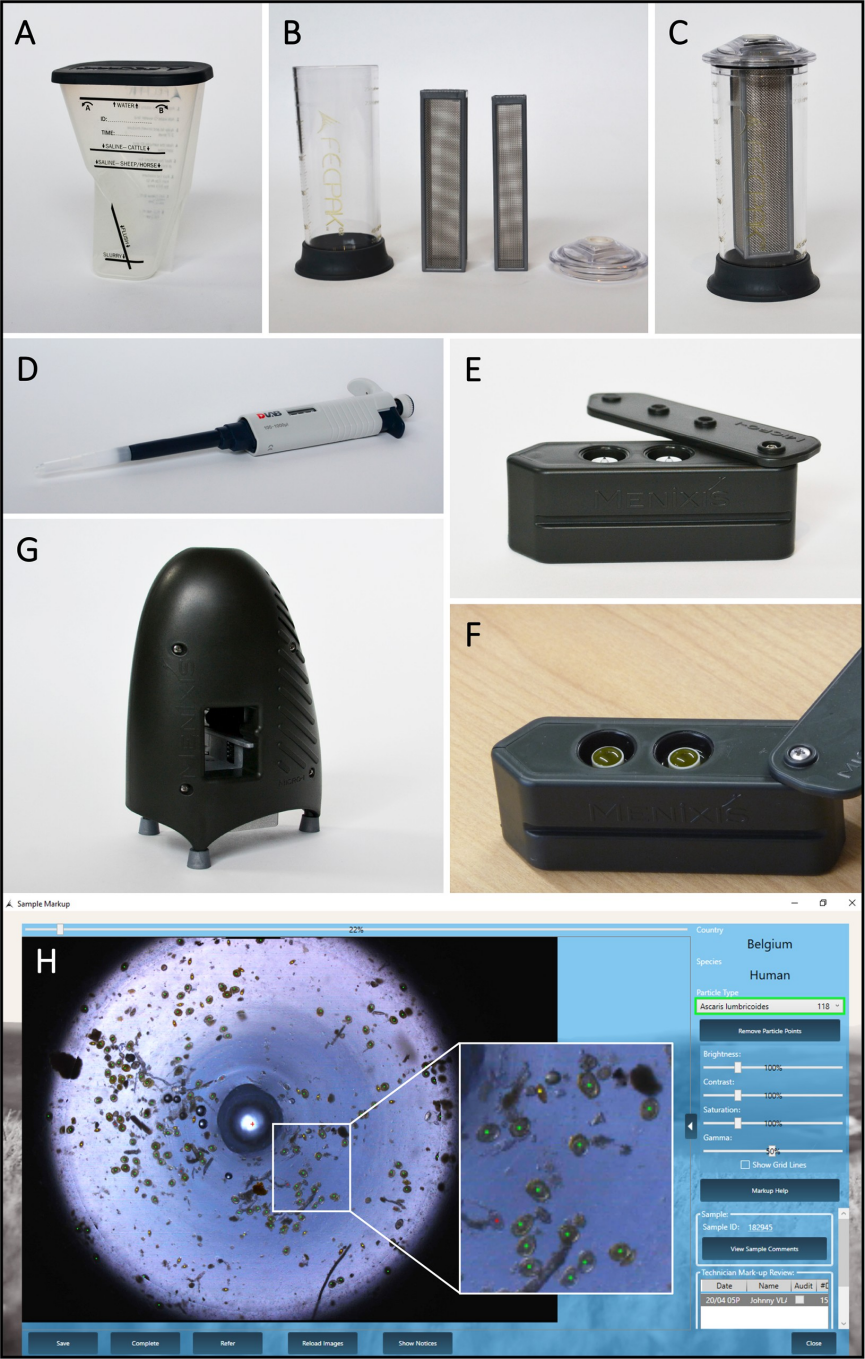
Materials needed to perform the FECPAK^G2^ method. (A) The FECPAK^G2^ sedimenter with indications of the water line and the saline lines used to analyse faeces of cattle or sheep and horses. For analysis of human samples, the saline line for sheep and horses is applied. (B) The filtration unit with cylinder base, cylinder top and two filter frames. (C) The unit fits together and can be disassembled for cleaning. The two filter frames have different mesh sizes (425μm and 250μm) and are designed to be used together. Samples are collected from the filtration unit using a pipette with special extension tip designed to fit into the filtration unit and the top of the cassette (D). The FECPAK^G2^ cassette with its two counting wells with central light rods is shown in side view (E) and from the top containing a sample (F). A filled cassette is fed into a portal of the MICRO-I device (G) and then pulled through automatically for imaging of each well. (H) A standard image that is produced by the FECPAK^G2^ and presented to the user. Some debris and numerous STH eggs are visible. Visible *Ascaris lumbricoides* eggs (n = 118) are marked with green dots, the *Trichuris trichiura* eggs are marked with yellow dots (n = 29) and the hookworm eggs are marked with a red dot (n = 5). After mark-up, this information is stored and remains available for review by authorized users.

**Fig 2:**
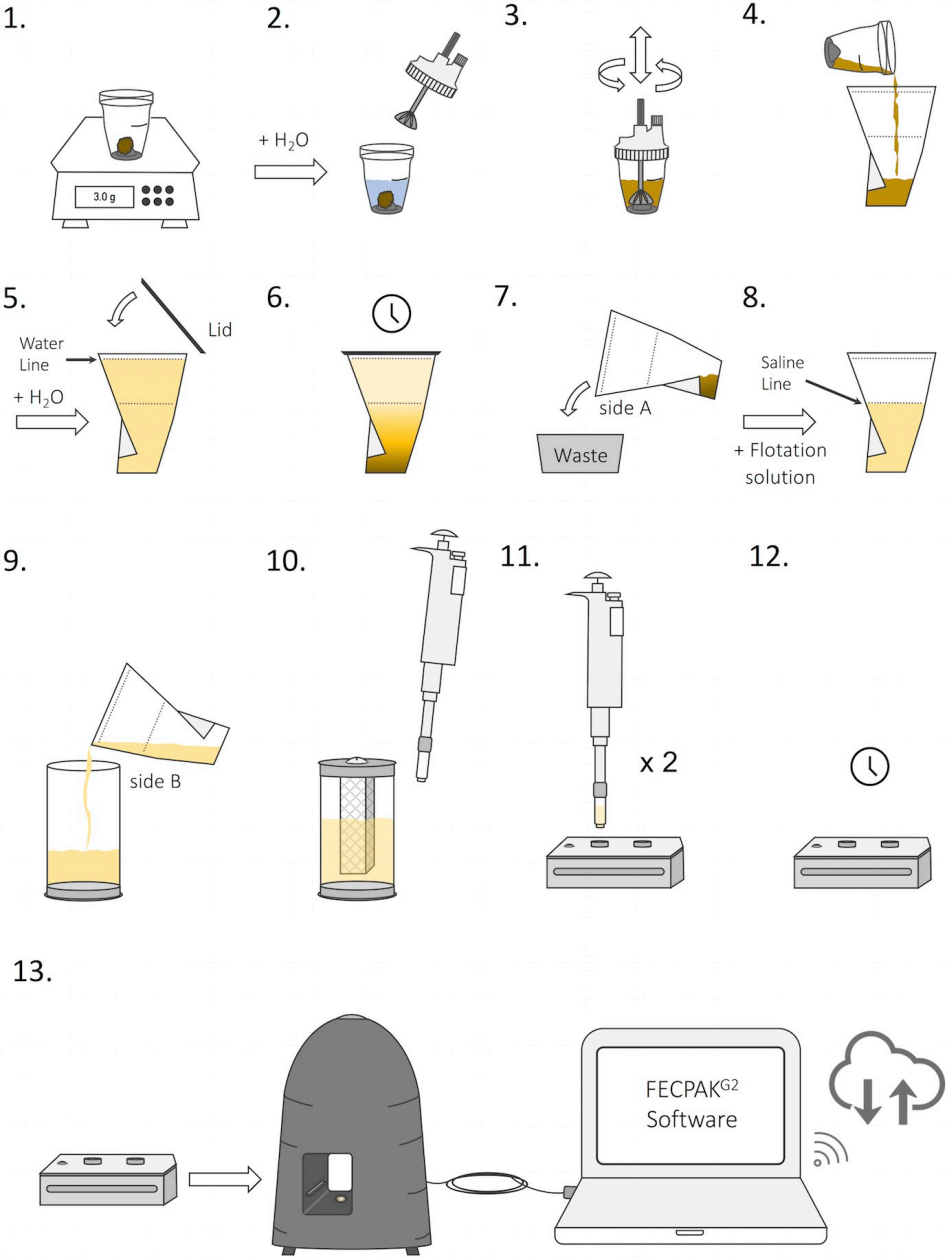
The FECPAK^G2^ standard operating protocol. Three grams of fresh human stool is weighed in a Fill-FLOTAC device (step 1), water is added and the stool is homogenized using the Fill-FLOTAC device (Step 2 & 3). The fecal suspension is then transferred to a FECPAK^G2^ sedimenter (step 4) and water is added to the water line (step 5 & 6). The supernatant is removed by tilting the sedimenter towards the A-side (step 7) and floatation solution is added to the retained slurry until the saline line is reached (step 8). This mixture is transferred into a FECPAK^G2^ filtration unit by pouring from the B-side of the sedimenter (step 9). The content is mixed by inverting the filtration unit 3 times and a sample is extracted from the inside of the inner sieve using a pipette with special tip. Samples are pipetted into both wells of a FECPAK^G2^ cassette (step 10 & 11). The filled cassette is then placed on a level surface to allow accumulation of the helminth eggs (step 12). Finally, the cassette is placed in the MICRO-I device and both wells are imaged (step 13). Images, together with accompanying sample information is stored and uploaded to the cloud.

Images of the key equipment needed to perform the FECPAK^G2^ method are shown in Fig 1, including the sedimenter, filtration unit, pipette, cassette, MICRO-I and software interface. A detailed schematic representation of the FECPAK^G2^ procedures for human stool is depicted in Fig 2. Stool analysis starts by weighing 3 grams of fresh stool in the container of a Fill-FLOTAC device (step 1). After addition of tap water to the 40-ml indicator on the Fill-FLOTAC (step 2), the stool is homogenized by pumping the conical collector up and down, while turning it to the right and left (step 3). After homogenization, the stool suspension is transferred to the FECPAK^G2^ sedimenter (Fig 1A, step 4). The Fill-FLOTAC device is rinsed with another 40 ml of tap water and this solution is added to the sedimenter as well. Finally, water is added to the water line, indicated just below the top of the sedimenter (approximately 210 ml) (step 5). The purpose of the sedimenter is to separate debris that floats in tap water from the helminth eggs and other debris that sediments in water. After closing the sedimenter with the lid, it is inverted three times to mix the solution and the sedimenter is then left undisturbed. This is called the sedimentation step (step 6). After sedimentation, the supernatant is carefully decanted off using the A-side of the sedimenter, hereby leaving the helminth eggs trapped in the bottom section of the sedimenter (step 7). A plastic dam prevents the remaining stool sediment or slurry (approximately 15 ml), containing the sedimented helminth eggs, from being discarded. The remaining slurry is subsequently mixed with flotation solution (FS) (saturated saline solution with a specific gravity of minimum 1.20 [16]). The FS is added until the saline line is reached (15 ml slurry + 80 ml FS = 95 ml) (step 8). The entire content of the sedimenter is then poured from the B-side of the sedimenter into the FECPAK^G2^ filtration unit (Fig 1C, step 9). The filtration unit consists of a transparent cylinder and two mesh filter frames that fit into each other and can be fixed on the inside of the lid (Fig 1B). The outer filter has a mesh size of 425 μm, while the inner filter has a finer mesh size of 250 μm. The filters help to further clean the sample using passive filtration. The filtration unit is closed and the sample mixed thoroughly by inverting the unit 3 times. The center of the lid holds an opening that is covered by a rubber valve through which aliquots of the sample are taken using a pipette with a specially designed extension tip (Fig 1D) to fill the two wells of the cassette (step 10). The cassette has two counting wells with central light rods (Fig 1E). Each well can hold 455 μl of sample. Two aliquots of 455 μl are taken from the filtration unit. Between aliquots, the filtration unit is again inverted three times to ensure the solution is mixed properly. The aliquots are pipetted into the two corresponding wells of the FECPAK^G2^ cassette through the opening in the cassette lid (step 11). The lid of the cassette is then turned open to visually ascertain that a proper fill level is reached (Fig 1F). This is a crucial step because wells that are overfilled or underfilled do not produce good images. After closing the lid of the cassette, it is left undisturbed to allow the helminth eggs to accumulate around the light rods (step 12). This is called the accumulation step. Finally, the cassette is inserted into the MICRO-I for imaging (step 13). The MICRO-I (Fig 1G) is an imaging device and connects to the FECPAK^G2^ software using a computer or tablet. When the cassette containing the sample is inserted into the MICRO-I it moves through automatically. The MICRO-I contains a light source and camera and takes a digital image of each well around the light rod where the parasite eggs have accumulated. This process takes approximately 5 minutes per cassette. After the imaging step, the cassettes are expelled from the MICRO-I and can be emptied and rinsed with tap water for reuse. Acquired images are stored and can be uploaded to the FECPAK^G2^ software when internet is available. The FECPAK^G2^ software stores the digital image alongside all the sample reference information (e.g. sample ID, sample collection date and study site) and enables trained technicians to view the uploaded image and identify and count the parasite eggs online. Results are stored in a cloud-based database which can be accessed by nominated and registered FECPAK^G2^—users.

### Optimization of the FECPAK^G2^ SOP for analyzing human stool

In order to optimize the FECPAK^G2^ SOP for the detection of STH eggs in stool, two crucial steps in the general SOP were evaluated. First, we investigated the time for optimal recovery of STH eggs in the slurry during the sedimentation step (step 6). Subsequently, the accumulation step was evaluated to determine the optimal time for human STH eggs to accumulate in the FECPAK^G2^ cassette (step 12).

#### Sample collection and selection

Stool samples were collected from school-aged children (aged 5 to 14 years) from Brazil, Ethiopia, Laos and Tanzania. Upon arrival in the laboratory, stool samples were homogenized thoroughly using a spatula. After this, samples were screened for the presence of STH eggs and the fecal egg counts (FEC; expressed as eggs per gram of stool (EPG)) for the different STHs were determined using a single Kato-Katz thick smear [17]. S1 Table summarizes the origin of each sample, their original FEC based on the single Kato-Katz thick smear and for which experiment they were used (optimal sedimentation/accumulation time).

#### Determining the optimal sedimentation time

**Fig 3:**
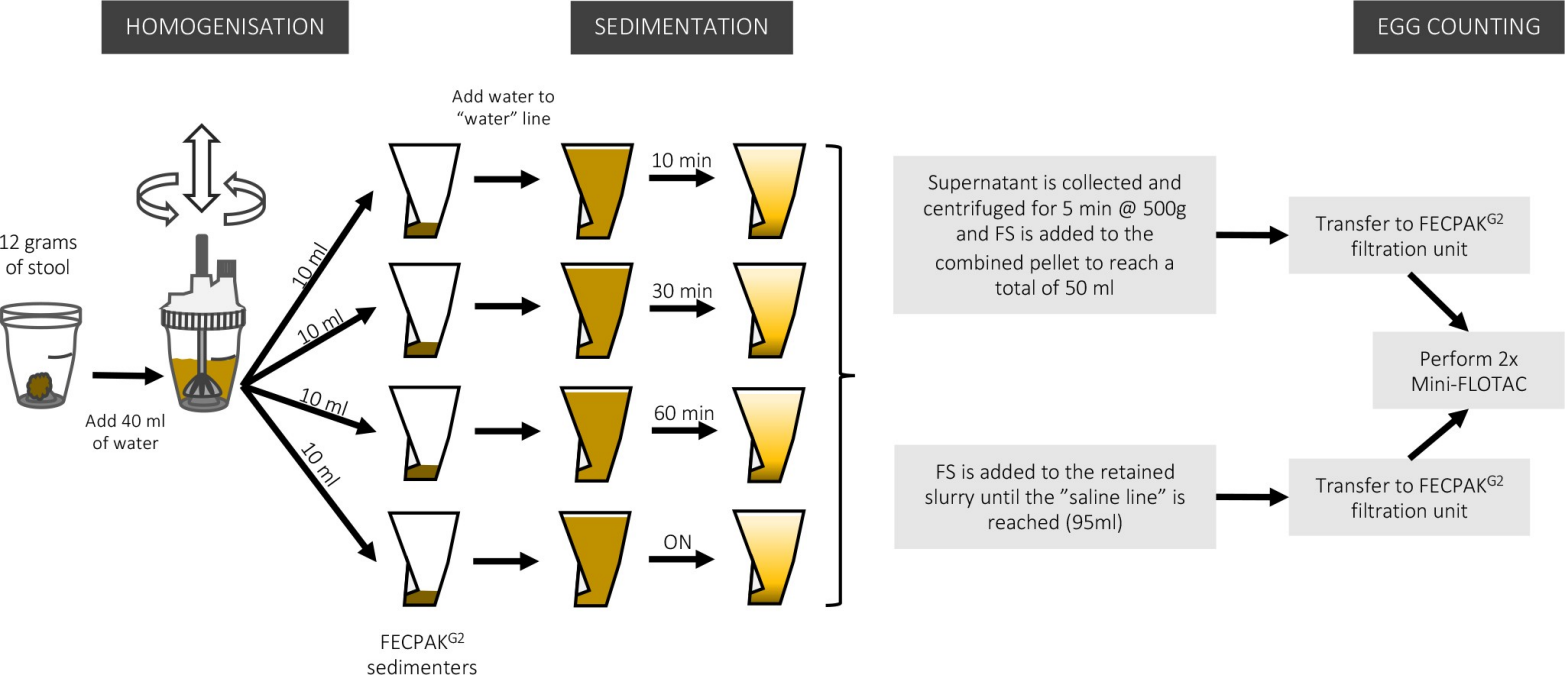
Schematic representation of the experiment designed to evaluate the optimal sedimentation time.

A schematic representation of this experiment is provided in Fig 3. To evaluate the effect of increasing sedimentation times, 15 stool samples from Ethiopia were processed using FECPAK^G2^ with 4 different sedimentation times for each sample. All samples contained at least 150 EPG for least one STH species. In short, 12 grams of stool (4 x 3 grams to distribute over 4 different sedimenters) was first homogenized in a Fill-FLOTAC device in 40 ml of water. Then, 10 ml of the obtained stool suspension was randomly divided over 4 different sedimenters (one for each sedimentation time: 10 min, 30 min, 60 min and overnight (ON)). All sedimenters were subsequently filled with tap water to the water line and left to sediment for 10, 30, 60 min or ON (steps 1–6).

After sedimentation, the supernatant of each sedimenter was poured off into a separate container. FS was added to the remaining slurry until the saline line (= total of 95 ml) was reached and the solution was subsequently transferred to a FECPAK^G2^ filtration unit (steps 7–10). Since the recovery rates that can be obtained with the FECPAK^G2^ for human STHs were unknown, the eggs in the FS were counted using an established flotation-based technique that is known to provide accurate and precise egg counts, namely the Mini-FLOTAC [15]. Solution was extracted from the FECPAK^G2^ filtration units to fill two Mini-FLOTAC devices (2 x 2 chambers of 1 ml). Between filling each of the 4 Mini-FLOTAC chambers, the solution in the filtration unit was homogenized by inverting the solution 3 times. The number of eggs was counted in both Mini-FLOTACs for each of the STH species separately. The total number of eggs in the filtration unit was calculated by multiplying the total number of eggs counted in the 2 Mini-FLOTACs by 24 (= total volume of suspension in filtration unit / total volume of suspension examined with Mini-FLOTAC = 95 ml / 4 ml).

To count the number of STH eggs that did not settle in the sediment, the supernatant was collected (approximately 200 ml) and transferred into four 50 ml tubes. These tubes were then centrifuged for 5 min at 500 *g* to pellet all possible STH eggs that were still present in the supernatant. After centrifugation, the obtained supernatant was discarded, and the pellets were combined and re-suspended in FS up to a final volume of 50 ml. This suspension was subsequently transferred into a second FECPAK^G2^ filtration unit, and 2 Mini-FLOTAC devices were filled with this suspension. Between filling each of the 4 Mini-FLOTAC chambers, the solution in the filtration unit was homogenized by inverting the solution 3 times. The total number of eggs in the filtration unit was calculated by multiplying the total number of eggs counted in the 2 Mini-FLOTACs (total volume examined is 2 x 2 chambers of 1ml = 4 ml) by 12.5 (= total volume of suspension in filtration unit / total volume of suspension examined with Mini-FLOTAC = 50 ml / 4 ml).

The egg recovery rate for each of the STH species was calculated for the different sedimentation times. The egg recovery rate (expressed in % of eggs recovered) was calculated using the following formula: Egg recovery rate (in %) = 100 x total number of eggs in slurry / total number of eggs in slurry + supernatant.

Egg recovery rates were only calculated for those STH species for which the FEC in a sample was higher than an average of 100 EPG after overnight sedimentation (sum of eggs detected in Mini-FLOTAC A+B ≥ 20). This was done to avoid the impact of the effect of samples with very low egg counts. Because in these samples, the presence of one egg potentially increases the recovery rate by more than 10%. Using these criteria, sedimentation data was available for 8 *Ascaris*, 11 *Trichuris* and 5 hookworm samples (S2 Table).

### Determining the optimal accumulation time

**Fig 4:**
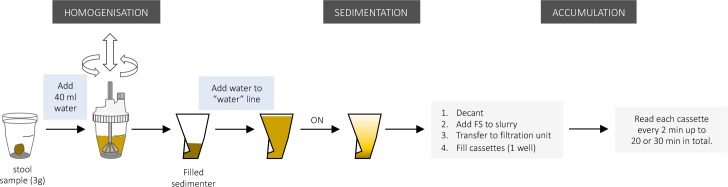
Schematic representation of the experiment designed to evaluate the optimal accumulation time.

A schematic representation of these experiments is provided in Fig 4. A total of 40 samples (= 4 countries x 10 samples) were prepared according to the adapted FECPAK^G2^ SOP for human STH (Table 1) with ON sedimentation. Briefly, three grams of stool from each sample was homogenized in 40 ml of water in a Fill-FLOTAC device and then poured into a sedimenter. Another 40 ml of water was used to wash the Fill-FLOTAC and was added to the respective sedimenters. Additional water was used to fill the sedimenters up to the water line before they were set aside for ON sedimentation (steps 1–6). The following day, the supernatant was discarded, and FS was added to the remaining slurry until it reached the ‘saline’-line (= total volume of 95 ml) (steps 7–10). This suspension was then transferred to a FECPAK^G2^ filtration unit and used to fill a single well of three different FECPAK^G2^ cassettes. Filled cassettes were entered in the MICRO-I, and FECPAK^G2^ software (‘FECPAK^G2^ Timed Capture’-software (Techion Group)) was used to capture images of the wells every 2 minutes for a total of 20 (Ethiopia). This preliminary data suggested that a longer accumulation time would be necessary to allow for complete accumulation of *Trichuris* eggs. Therefore, the accumulation time was extended to 30 minutes for the other 3 countries where we performed the experiment (Brazil, Laos and Tanzania). After taking the first set of images (approximately 2 minutes after filling the wells), the cassette stayed in place to prevent movement of the accumulated eggs by moving the cassette inside the MICRO-I device. Later, the total number of STH eggs across the three images was counted at different time points over the course of the accumulation period. For each STH species, the sum of the number of eggs counted in the 3 cassettes at each time point was divided by the maximum number of detected eggs for that STH species over any of the time points to obtain a relative recovery percentage. To ensure the production of representative accumulation curves for the different STH eggs, samples that did not show 10 detectable eggs over the 3 wells combined at any measured time-point were excluded from downstream analysis.

#### Statistical analysis

Significant changes in egg recovery rates over increasing sedimentation times were indicated by one-tailed paired t-tests. Level of significance for each of the tests was set at *p* <0.05. All statistical analyses were performed using GraphPad Prism v6.0 software (La Jolla, CA, USA).

## Results

### Determining the optimal sedimentation time

**Fig 5:**
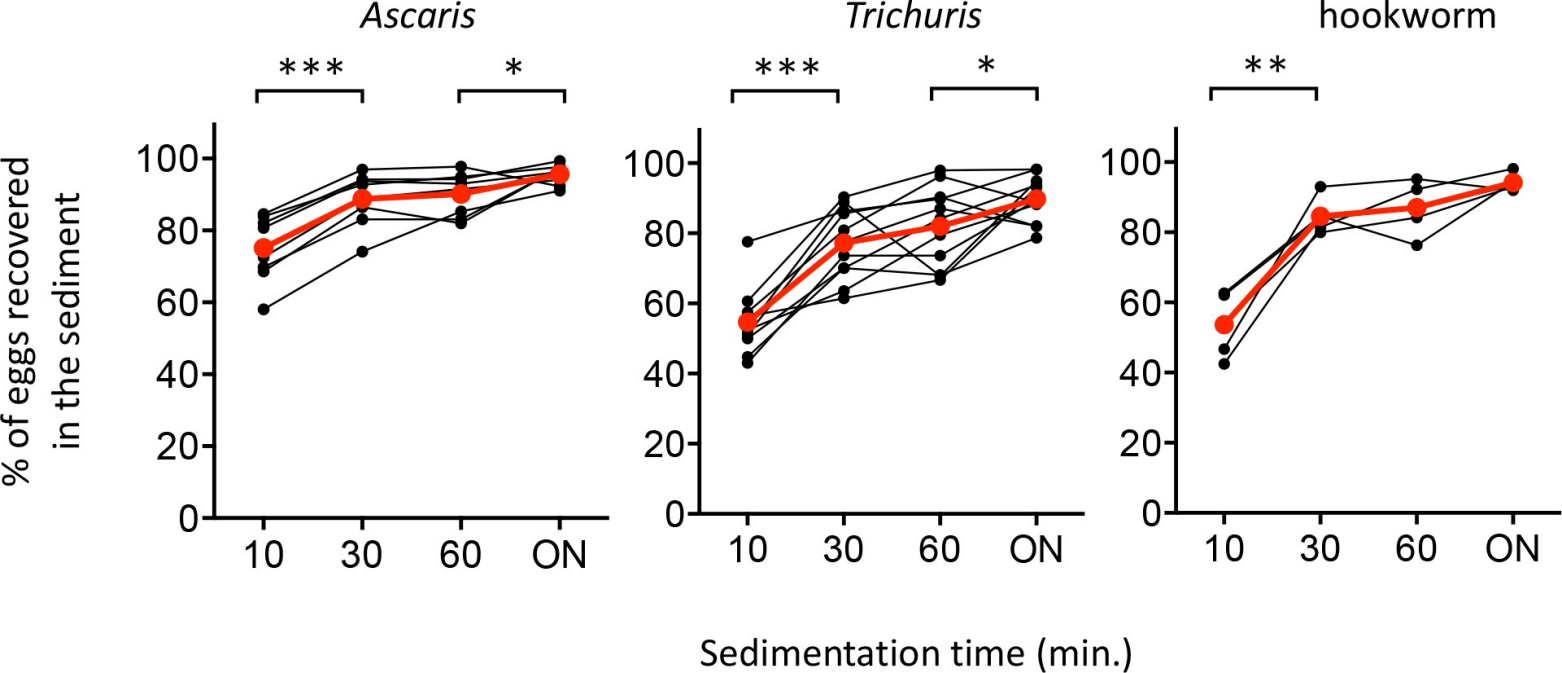
Line plots representing the percentage of STH eggs recovered in the slurry of the sedimenter at different sedimentation times (10, 30, 60 minutes and overnight (ON)). The average recovery percentage over the different samples is indicated in red. Significant increases in egg recovery percentages between subsequent sedimentation times are indicated (* P < 0.05; ** P < 0.01, *** P < 0.001).

A total of 15 stool samples from Ethiopia were included in this experiment (S1 Table). The egg recovery rates, expressed in percentages of the eggs recovered from the slurry compared to the total amount of eggs recovered (slurry + supernatant) after different sedimentation times are presented in Fig 5. The raw egg counts in the Mini-FLOTAC, the total egg counts per fraction, and calculations of the recovery rates are provided in S2 Table. For each of the STH species, there was an increase in egg recovery rate over time with ON sedimentation resulting in a maximum recovery rate. However, the increase in egg recovery rate over time was not consistent across the different STH species. For *Ascaris* (n = 8), the mean (±SD) egg recovery increased significantly from 75.1% (± 9.4) after 10 min to 88.8% (± 7.5) after 30 min (*p* <0.0001), and then stayed more or less unchanged after 60 min (90.2% ± 6.0) sedimentation. Overnight sedimentation further increased the recovery percentage to 95.7% (± 2.9) (*p* = 0.0298). For *Trichuris* (n = 11), the mean egg recovery significantly increased from 54.7% (± 9.2) after 10 min of sedimentation to 77.2% (± 10.6) (*p* < 0.0001) and 82.0% (± 11.5) after 30 or 60 min of sedimentation, respectively. Recovery rates further increased and reached a maximum of 89.8% (± 6.6) (*p =* 0.0304) after ON sedimentation. The mean egg recovery rates for hookworm eggs (5 samples) was 53.7% (± 9.0) after 10 min of sedimentation. This significantly increased up to 84.6% (± 2.1) (*p* = 0.0017) when samples sedimented for 30 min. After 60 min of sedimentation, there was an insignificant increase to 87.0% (± 7.4). Hookworm egg recovery rates eventually reached a maximum of 94.2% (± 2.4) (*p* = 0.0514) after ON sedimentation. A sedimentation time of minimum 1 hour is thus needed to be able to recover a minimum of 80% of the eggs for all three STH species.

### Determining the optimal accumulation time

**Fig 6:**
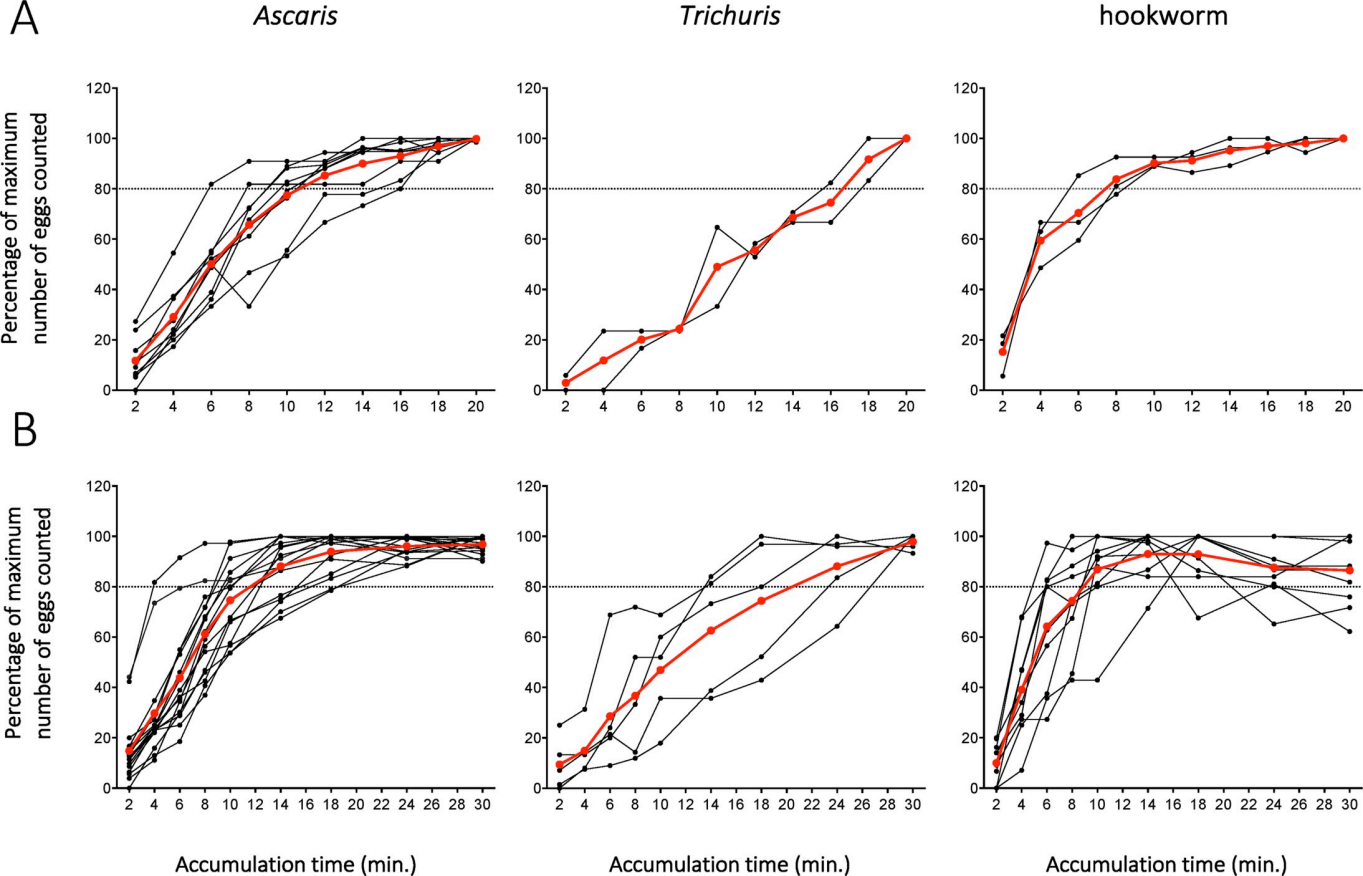
Line plots showing the average relative amount of STH eggs counted in FECPAK^G2^ cassettes over increasing accumulation times. The average relative percentage of visible eggs in the different samples over the different time points is indicated in red. Panel A shows the results obtained from the accumulation experiment performed with 10 samples from Ethiopia where samples were imaged for 20 minutes. Panel B shows the combined results obtained from the accumulation experiment performed with 30 additional samples (10 from Brazil, 10 from Laos and 10 from Tanzania). These samples were imaged for a total of 30 minutes. A grey horizontal dotted line is drawn at the height of 80% of maximum number eggs counted in the cassette wells.

To examine the optimal accumulation time for the different STH eggs in the FECPAK^G2^ cassette, 40 samples containing STH eggs were processed using the general FECPAK^G2^ SOP with ON sedimentation from 4 different endemic countries (10 samples x 4 countries). The raw data of the egg counts and the percentage of eggs accumulated per sample are provided in S3 Table. This experiment was first performed in Ethiopia using 10 selected samples, of which 9, 2 and 3 samples could be used to produce accumulation curves for *Ascaris*, *Trichuris* and hookworm eggs, respectively. The relative recovery percentages for each of the STH species observed in the Ethiopian samples are plotted over time in Fig 6A. This experiment revealed that the different STH species show different accumulation curves in FS inside the wells of the FECPAK^G2^ cassette. Hookworm eggs appeared to accumulate the fastest in the FECPAK^G2^ cassette wells, with (83.8% ± 7.8) of the maximum number of eggs accumulated in the wells after 8 minutes of accumulation. For *Ascaris* eggs it took 12 minutes to reach over 80% of the maximum number of eggs counted in the wells (85.3% ± 8.6), after which the number of eggs in the wells continued to increase slowly towards 99.8% ± 0.5 at 20 minutes of accumulation. In contrast, the number of *Trichuris* eggs counted in the wells of the 2 samples increased more steadily over the 20 minutes of accumulation time. Only after 18 minutes, more than 80% of the total number of eggs (91.7% ± 11.8) had accumulated in the wells. Moreover, the accumulation curves did not reach an apparent plateau as was seen for *Ascaris* and hookworm samples.

Due to the limited number of samples examined in Ethiopia, in combination with the apparent finding that, especially for *Trichuris*, an accumulation time longer than 20 minutes was needed, this experiment was repeated with 3 x 10 samples from 3 other countries and the accumulation time was extended to 30 min. For these 30 samples, the pictures taken after 2, 4, 6, 8, 10, 14, 18, 24 and 30 minutes of accumulation time were examined. This resulted in a combined total of 16 additional *Ascaris* samples (Brazil: 9, Laos: 5, Tanzania: 2), five *Trichuris* samples (Laos: 1, Tanzania: 4) and 11 hookworm samples (Brazil: 1, Laos: 7, Tanzania: 2) (S3 Table). The accumulation curves obtained by analyzing the samples from Brazil, Laos and Tanzania are shown in Fig 6B. These results confirmed the results obtained with the Ethiopian samples. Again, hookworm eggs accumulated rapidly in the wells, reaching a recovery rate higher then 80% (86.9% ± 17.1) by minute 10. The maximum number of hookworm eggs (93.1% ± 9.6) were visible after 14 minutes, after which accumulation percentages remained stable or even slightly, but insignificantly, decreased again to 92.9% (± 10.9), 87.6% (± 11.0) and 86.5% (± 13.4) at 18, 24 or 30 minutes of accumulation time respectively. The number of *Ascaris* eggs that accumulated in the wells reached an average of 88.1% ± 11.7 after 14 minutes of accumulation. This continued to increase minimally to a recovery of 96.8% ± 3.4 at the end of the measured accumulation period. For *Trichuris*, the average percentage of visible eggs increased notably slower than for the other two helminth species. A recovery of 88.2% ± 14.7 of the maximum average egg count was noted after 24 minutes of accumulation. The average accumulation percentage further increased to 97.9% (± 3.1) at 30 min of accumulation. However, this increase was not significant. An overall accumulation time of at least 24 minutes is thus needed in order to allow accumulation of at least 80% of the eggs for all three STH species.

## Discussion

The FECPAK^G2^ is a diagnostic tool that was originally designed for the diagnosis of helminth infections in sheep and cattle [11]. The present study evaluated two important steps in the FECPAK^G2^ SOP. Both the optimal sedimentation time in the FECPAK^G2^ sedimenter and the accumulation time in the FECPAK^G2^ cassettes of human STH eggs were determined.

Although more than 80% of the eggs of all three STH species had sedimented after 1 hour of sedimentation, the highest egg recovery rate was observed after ON sedimentation. With exception of hookworms, with a borderline significance, the ON sedimentation time provided significantly higher egg recovery numbers compared to 1 hour sedimentation. However, recommending ON sedimentation in the FECPAK^G2^ SOP for detecting STH eggs in human stool has some important practical implications that may not be ideal in a programmatic setting. First, ON sedimentation would not allow for high throughput, as it delays processing of the samples. Second, this approach requires a sufficient number of sedimenters so that all samples collected in one day can be processed, which would require sufficient lab space. Finally, ON sedimentation should ideally happen in a cooled or acclimatized environment since Udonsi and Atata [18] showed that *N*. *americanus* larvae can hatch within 24h after excretion when stool is stored at a temperature of 25˚C or higher. As a consequence of larval hatching, egg counts might be biased. A thorough comparative analysis to evaluate the effect of ON incubation in uncontrolled, elevated ambient temperatures on the recovery rates of hookworm eggs was not performed in this study. Nevertheless, in Laos, where ON sedimentation did not occur in an acclimatized laboratory setting, a preliminary test with 5 samples containing hookworm eggs did not reveal significant differences in recovery rates obtained by FECPAK^G2^ after 60 min or ON sedimentation (S1 Data).

The way particles like helminth eggs behave in water is expected to follow Stoke’s law, implying that settling velocity depends on particle size, density and water viscosity. *Trichuris* eggs, which are the densest STH eggs (1.15 g/cm^3^), are thus expected to settle faster in water than either *Ascaris* (1.11g/cm^3^) or hookworm eggs (1.055g/cm^3^), which have a lower density [19]. Sengupta et al., [20] showed that this is largely true when eggs are settling in tap water, but that the settling velocities of eggs changed significantly in wastewater. The average settling velocity of *Ascaris* eggs sharply increased from 0.06 mm/s in tap water to 0.16 mm/s in wastewater, while the average settling velocity of *Trichuris* eggs decreased from 0.15 mm/s to 0.09 mm/s. These results correspond with the findings of our sedimentation experiment, where we also noticed a slower increase in number of *Trichuris* eggs in the sediment compared to the eggs of the other two parasite species. A high concentration of particles in wastewater typically results in flocculation, a process whereby particles in a solution form larger-sized clusters or flocs due to contact and adhesion forces [21].

The second important optimization step of the FECPAK^G2^ SOP was the accumulation step. We found that an accumulation time of minimum 24 minutes is necessary to allow accumulation of at least 80% of the total recoverable eggs coming for each of the STH species. This is significantly longer than 6 min, which is the current recommended accumulation time for veterinary parasite eggs [11]. But this is largely due to *T*. *trichiura*, which shows notably slower egg accumulation in the cassettes and is thus the time limiting species in this step. The optimal accumulation time for human STH was unknown until now. It is however a crucial aspect of the FECPAK^G2^ protocol. Taking the pictures too early after filling the wells might lead to systematic lower egg counts and a higher number of false negative results. On the other hand, waiting too long to capture the images could similarly affect egg counts. First of all, a longer accumulation time favors the crystallization and/or evaporation of the saline solution resulting in FECPAK^G2^ images of inferior quality, which, in its turn, leads to difficulties identifying the eggs during mark-up. Second, in a number of samples containing hookworm eggs, the egg counts decreased over time after reaching a maximum at 14 minutes of accumulation. The reason for this is unknown, but it is possible that prolonged presence in a hypertonic saline solution slowly increases the density, and thus decreases buoyancy of the hookworm eggs as a result of fluids being extracted from the egg through the egg shell. This is supported by Matthews [22], who also noted that eggs of *Ancylostoma caninum* were permeable and susceptible to osmotic changes early in their development. This issue is likely to be less of a problem for *Ascaris* and *Trichuris* given their thicker egg shell. For these latter two parasite species, the accumulation curves remain largely horizontal once the maximum number of visible eggs is reached. Although this technology would allow to take images at different time-points of accumulation that are optimal for each particular worm species, this would significantly complicate the technique.

The steepness of the accumulation curves that were produced by our experiments reflect the speed at which eggs accumulate around the tip of the rod on the top of the flotation solution surface. This is also affected by the specific physical properties of the eggs. The accumulation curves produced by *Trichuris* eggs, which are the densest STH eggs (1.15 g/cm^3^), was notably more gradual than the curves produced by either *Ascaris* (1.11g/cm^3^) or hookworm eggs (1.055g/cm^3^) (Fig 5B). The difficulties in floating *Trichuris* eggs could furthermore be affected by a larger variation in egg density as compared to *Ascaris* or hookworm-type eggs [20].

This study has some important limitations which stem from the fact that we were restricted to using stool samples from naturally infected individuals for our experiments. As a first consequence of this, the true egg counts remain unclear. This impedes the accurate calculation of the recovery percentages and hampers the evaluation of the repeatability and variation introduced by the different steps included in this method. A second consequence was that we were constrained to the naturally occurring infection prevalence and intensities. This resulted in a rather limited number of *Trichuris* samples (n = 5) being included in the accumulation experiments since *Trichuris* was either absent (Brazil) or present in very low prevalence and intensities (Laos) in the tested individuals. A future experiment in which we artificially spike negative stool samples with known numbers of STH eggs could remedy these shortcomings.

### Conclusion

In this work, we presented a SOP for the FECPAK^G2^ to be used for the detection of STH eggs in human stool samples. We also evaluated the two most important steps of the FECPAK^G2^ protocol to further optimize the method for the detection of human STH eggs. We can conclude that a sedimentation step of at least 60 minutes is necessary to recover a minimum of 80% of parasite eggs from the sample and that cassettes should be imaged no sooner than 24 minutes after filling to ensure a recovery exceeding 80% for all three types of STH eggs. Now that these important steps are validated, additional evaluations of the FECPAK^G2^ system are warranted. It will be necessary to evaluate its diagnostic performance and to compare its time and cost efficiency compared to other existing diagnostic methods. In a recent study, Moser and colleagues [23] already evaluated the FECPAK^G2^ system compared to the Kato-Katz method in the framework of a randomized controlled trial. FECPAK^G2^ showed a considerable lower sensitivity to detect any STH infection compared to Kato-Katz, particularly at low infection intensities. Further research is required to increase sensitivity and egg recovery so that FECPAK^G2^ can soon be added to the small number of available diagnostic tools for the microscopic detection of STH infections. A detailed SOP for the FECKPAK^G2^ technique is provided as supplementary data (S1 Text).

## Supporting information

S1 Table An overview of the samples included in the two experiments with their fecal egg counts as determined by single Kato-Katz. (XLSX)

S2 Table The number of STH eggs counted by two Mini-FLOTACs in the slurry and supernatants of 15 stool samples after 10, 30, 60 minutes or overnight sedimentation. (XLSX)

S3 Table The number of STH eggs counted in each of the pictures taken by the MICRO-I device over 20 or 30 minutes of accumulation time. (XLSX)

S1 Data Results of a preliminary experiment performed on 5 samples in Laos where the recovery rate for hookworm eggs was compared after 1 hour and overnight sedimentation. (XLSX)

S1 Text A detailed SOP of the FECPAK^G2^ technique for the detection of soil-transmitted helminth eggs. (PDF)
