# Correction: Modification and optimization of the FECPAK^G2^ protocol for the detection and quantification of soil-transmitted helminth eggs in human stool

**DOI:** 10.1371/journal.pntd.0007224

**Published:** 2019-03-04

**Authors:** Mio Ayana, Johnny Vlaminck, Piet Cools, Shaali Ame, Marco Albonico, Daniel Dana, Jennifer Keiser, Helen Manly, Leonardo F. Matoso, Zeleke Mekonnen, Antonio Montresor, Rodrigo Correa-Oliveira, Laura Rinaldi, Somphou Sayasone, Stephen J. Sowerby, Lensa Tesfaye, Jozef Vercruysse, Greg Mirams, Bruno Levecke

There are several errors in the article.

The fifteenth author's name is spelled incorrectly. The correct name is: Stephen J. Sowerby.

There is an error in the affiliation for author Stephen J. Sowerby. The affiliation should be: Department of Biochemistry, University of Otago, Dunedin, New Zealand.

There is an error in the Competing Interests section. The correct Competing Interests should read: The FECPAKG2 technology was produced and distributed by Techion Group Ltd, of which HM is an employee, and GM is a Managing Director. GM and SSo hold stock in Techion Group Ltd. These affiliations however did not play any role in the preparation and submission of this manuscript. All other authors declare that they have no competing interests.
