# Supplementary material for: Modification and optimization of the FECPAKG2 protocol for the detection and quantification of soil-transmitted helminth eggs in human stool
Source: PLoS Negl Trop Dis. 2018 Oct 15;12(10):e0006655. doi: 10.1371/journal.pntd.0006655 (PMC6224113; doi:10.1371/journal.pntd.0006655)
Supplement: S1 Text — (PDF) [file pntd.0006655.s005.pdf]

Authors:
Piet Cools, Bruno Levecke and
Johnny Vlaminck

FECPAK^{G2} Standard Operating Protocol.

1. Purpose

The FECPAK^{G2} has been launched as a complete remote-location diagnostic tool for sheep/cattle farmers and their veterinarians to assess both intensity of helminth infections and efficacy of drugs. The FECPAK^{G2} platform contains a cassette that concentrates helminth eggs into one microscopic field of view. Subsequently, the Micro-I photographs this view and stores it on a tablet or computer until internet is available. Later, a web-based lab technician can perform an egg count on the images, after which the results are returned to the user by e-mail and stored online for quality control, analysis, future reference and reporting. Since the similarities in egg morphology and the way efficacy of drugs are reported this platform also holds promise for soil-transmitted helminths in humans. This SOP describes the procedures to take and to mark-up the images.

2. Equipment

- Fill-FLOTACs

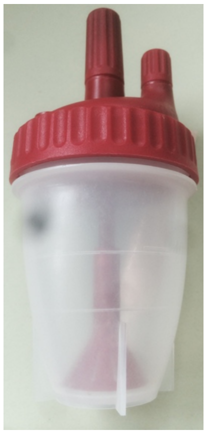

- FECPAK^{G2} sedimenters

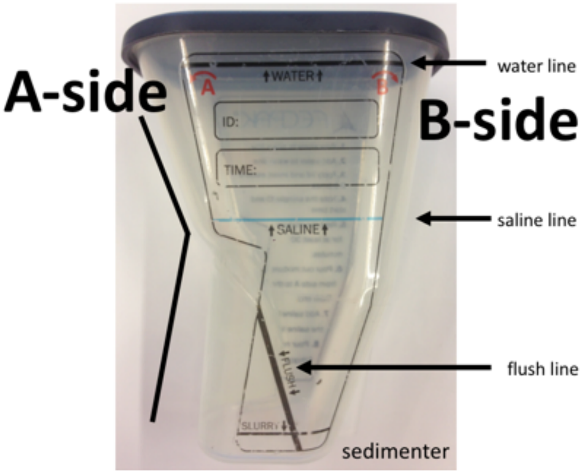

- FECPAK^{G2} filter cylinders

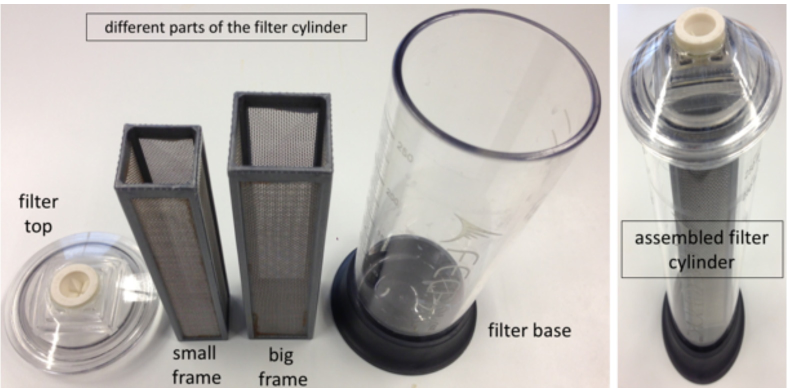

- FECPAK^{G2} cassettes

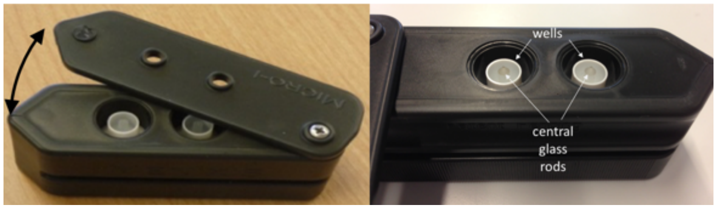

- 1 Micro-I

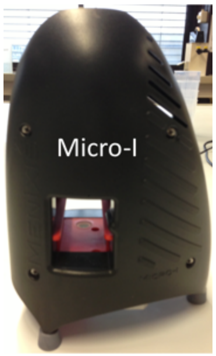

- Beaker with tap water

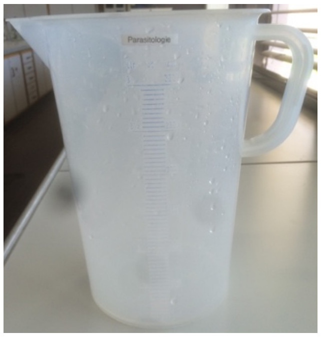

- Beaker with saline

(see SOP 03: Preparation of flotation solution)

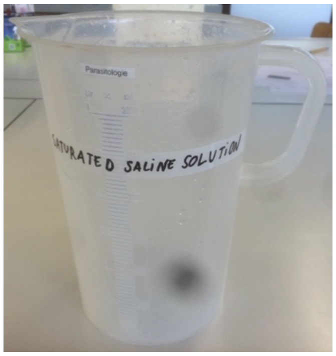

- Timer

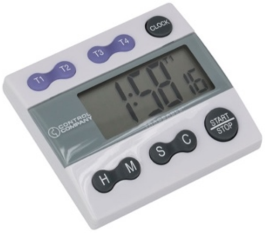

- Paper tape
- Computer with FECPAK^{G2} software
- Marker
- Weighing scale (0.1 g precision)
- Tissue paper
- Brushes to clean
- 0.5% Virkon solution
- Cotton swabs

3. Procedures

3.1 Preparation of samples up to the sedimentation step.

1. Place the Fill-FLOTACs next to each of the ordered samples.

2. Paste some paper tape on each of the Fill-FLOTACs and write the identification number corresponding to the sample on it. Make sure the paper tape is not fully taped on the stool container, in that way it can easily be transferred in downstream steps.

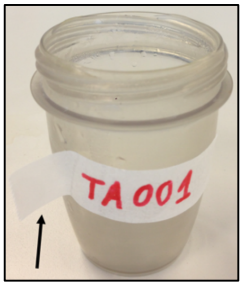

3. For each sample, place the Fill-FLOTAC container on the balance, press 'tare' and weigh **exactly 3.0 g** of stool using a spatula.

4. Add tap water from the beaker into the container to the 38 ml gradient line and close the Fill-FLOTAC.

5. Homogenize the sample by moving the applicator up and down while rotating, until the sample is fully homogenized.

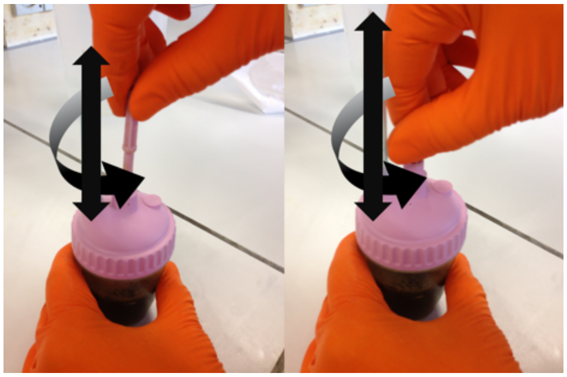

6. Open the Fill-FLOTAC and pour the suspension in the sedimenter. Rinse the container once with tap water and pour in the sedimenter.

7. Transfer the paper tape with the ID from the Fill-FLOTAC to the corresponding sedimenter as illustrated by the figure below.

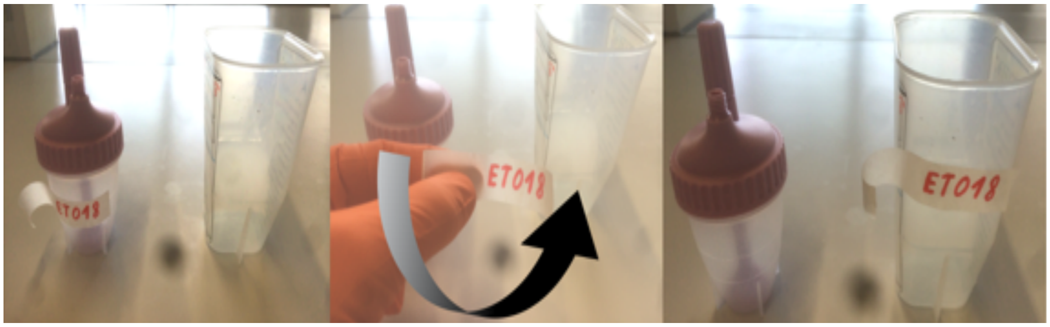

8. Add water to the sedimenter until the 'water line' is reached and close the lid.
   <u>Caution:</u> Make sure the lid is firmly closed on all four edges (you can hear it click when it is properly closed).
9. Invert the sedimenters 5 times and allow the samples to settle by keeping them on the bench at room temperature.
   <u>Caution:</u> Keep the sedimenters out of direct sunlight!
10. Allow the eggs and debris to sediment in the sedimenters for **at least 1 hour**.

3.2. Preparation of samples after the sedimentation step

1. After the sedimentation step, which should be at least 1 hour, slowly pour out the mixture from the sedimenter in ONE FLUID MOTION from side A until the flush line is in a HORIZONTAL position. Pour out in a beaker.

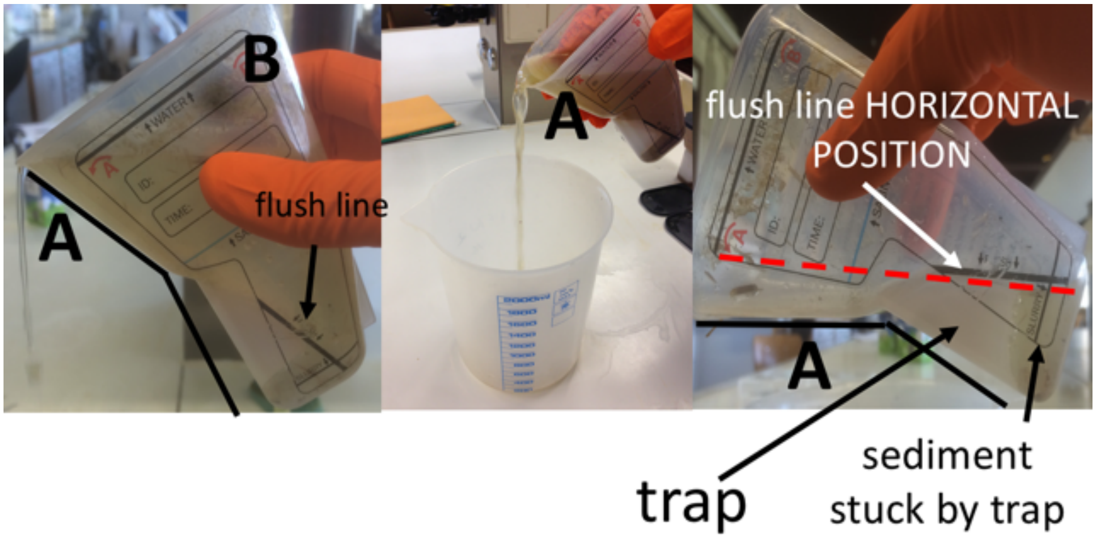

Caution: It's important to pour out in one fluid motion in order to avoid resuspension of the eggs.
Caution: It's important not to walk around too much with the sedimenter in order not to disturb the sedimented eggs. Therefore, it is important to pour out in a beaker that you can easily place on the bench where the sedimenters have stand. This is contrast when you walk with the sedimenters to the nearest sink.
Note: The sedimenter contains a trap on the bottom on the 'A' side (see Picture above). When pouring out the suspension from the 'A' side, the sediment, including the helminth eggs, will be withheld by the trap.

2. Add saline to the blue 'saline line' on the sedimenter.

3. Pour the mixture from side B of the sedimenter to the filter cylinder.

   Note: In this step, the sedimented helminth eggs can be poured out in the cylinder, so there is no need to pour out from the A side containing the trap. Resuspend when material remains in the sedimenter.

4. Transfer the label from the sedimenter to the filter cylinder and insert the filter into the cylinder.

5. Invert the mixture 3 times while closing the rubber opening with one finger.

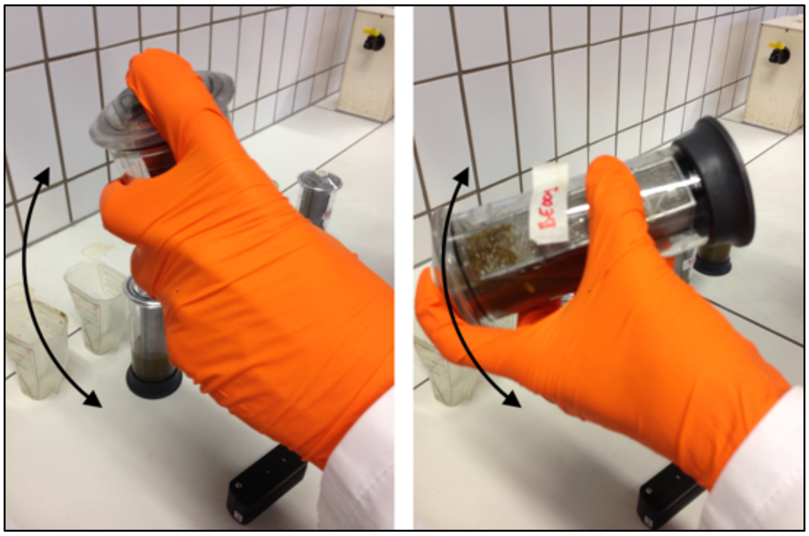

6. Promptly put a tip on the pipette with of volume of 455 µl and press the pipette button down to the second stop. Put the pipette into the sample through the rubber opening and aspirate the suspension by **gently** releasing the button fully until the rest position. <u>Caution</u>: This volume of 455 µl may vary across cassettes.

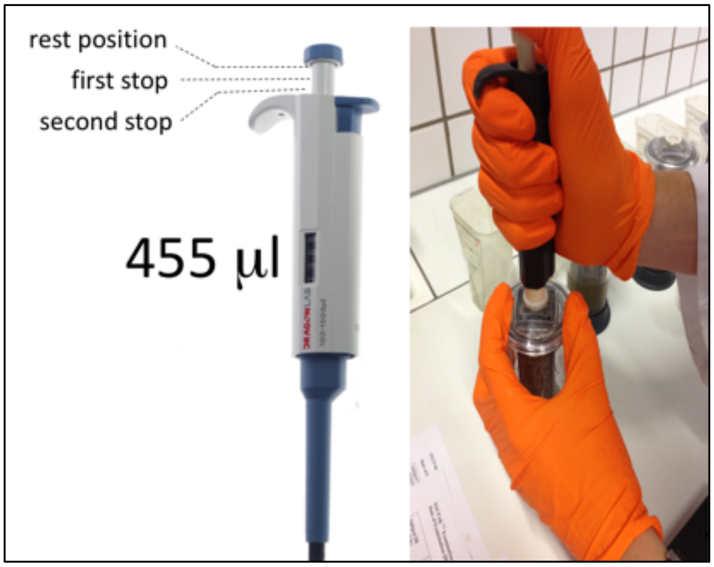

7. Take out the pipet from the filter cylinder and wipe the tip with tissue paper and place the pipette in the hole of the cassette.

   Caution: Do not hesitate too long during this step and the following step as eggs will start to float in the aspirated solution!

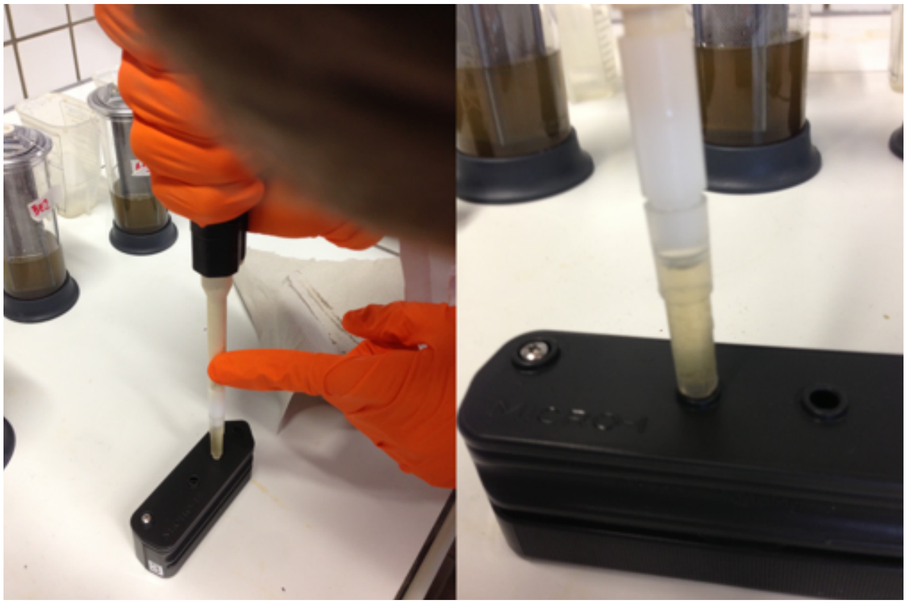

   Note: The pipet might struggle in the rubber opening when taking out. If the pipet tips is released and is lost in the filter container, open the filter container and take the pipet tip out. Restart at step 5.

8. Gently press the button down to the first stop to fill the first well.

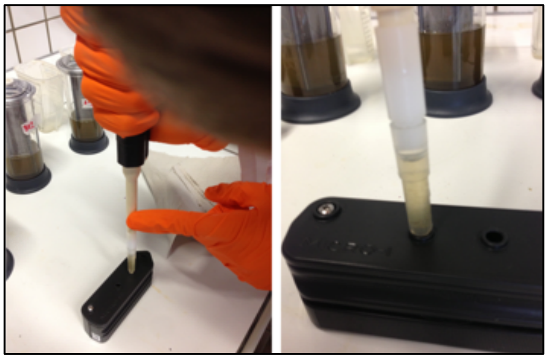

9. Take the pipet out and expel the remain of the suspension back into the cylinder by pressing in the pipette to the second stop. Hold the pipette in this position.

10. Check the fill. This is a crucial step, if not full enough or too full, the eggs will not be in a

good position for image capture. The rod of the well should be visible as depicted below, if not, the cassette has to be washed and refilled.

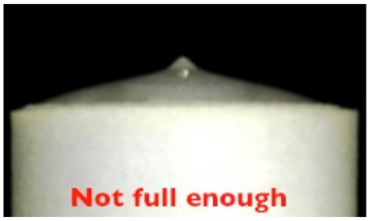

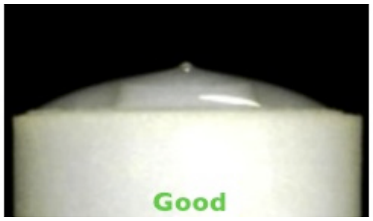

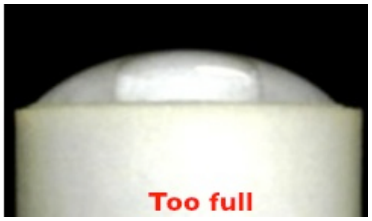

11. Invert the cylinder **3 times**. Fill the second well of the cassette by repeating steps 5–10. If the fill of the second well is good, close the cassette. If the fill of the second well is not satisfactory, wash the cassette and start again with **step 6**. Do not forget to invert the cylinder 3 times!
12. Paste a second tape on the cassette and write the participant ID on it.

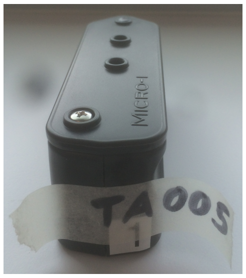

<ins>Note</ins>: when during image capture, if an unsatisfactory image is obtained from a cassette, this cassette needs to be refilled, and a new image needs to be captured. Therefore, it's important to know from which filter cylinder the cassette was filled, and both the filter cylinder <ins>AND</ins> cassette need to be labelled.

13. After filling the <ins>FIRST</ins> cassette, set a timer for **24 minutes** and allow the eggs to accumulate before image capture.

3.3 Submission of samples to the FECPAK^{G2} software

1. Initiate the FECPAK^{G2} Lab-Lite software by clicking on the desktop icon.

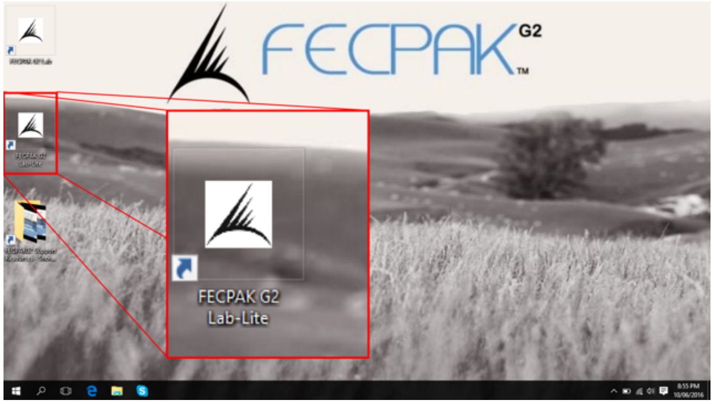

2. A login screen opens: fill in login, password and click on login.
3. A window opens with three options: ‘New submission’, ‘View/edit test’ and ‘Synchronise’.

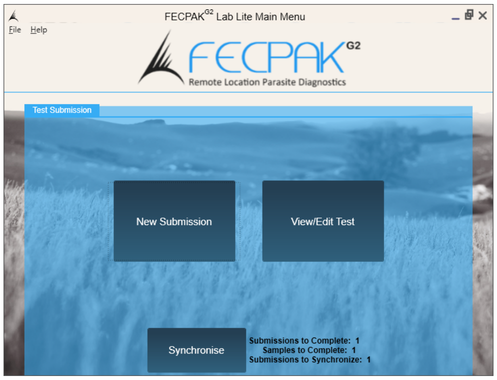

4. To add a new submission, click on ‘New Submission’. A new window opens. Select the name of your site and click on ‘select’ (you can also double click on your site name).

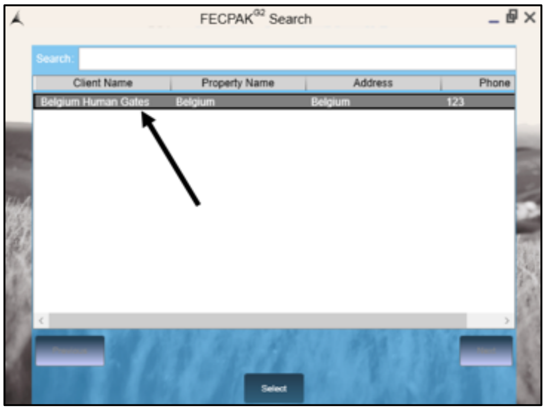

5. A window ‘New Test Submission’ opens. You do not need to change any settings. Just continue by clicking on ‘Save’.

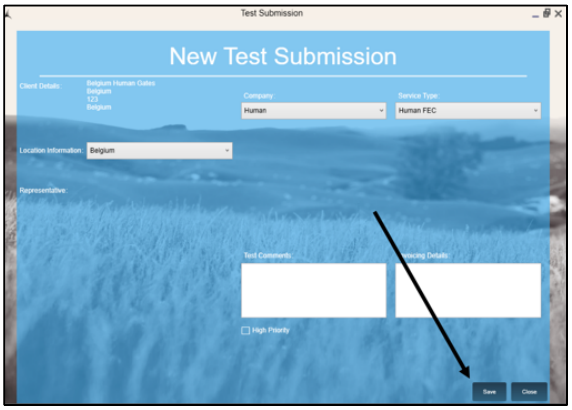

6. A window ‘Test Submission Details’ appears. To add data of a new subject, click on ‘Add Sample’.

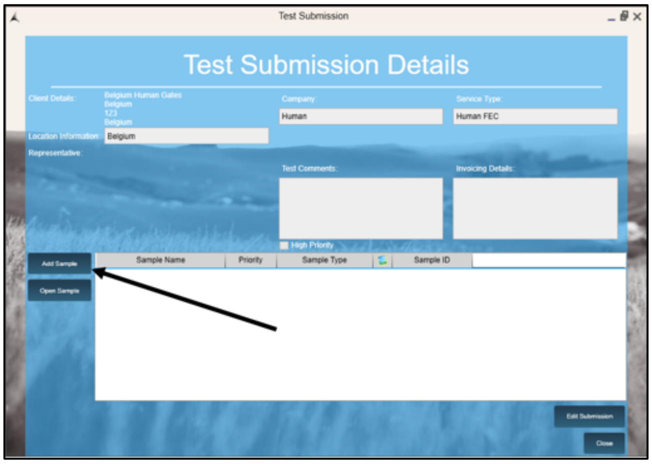

7. A new window opens in which different fields have to be completed like the Participant ID, age, sex, etc.

8. After completing all the fields, click on 'Save and Add New' to start a new sample submission. After completing all entries, click on 'Save and Close'.

3.4. Image capture

1. Place the Micro-I on a stable table. Make sure no other people are working on this bench or table. Make sure no apparatuses that might cause vibrations, e.g. centrifuges, are placed on the same bench or table.

   Note: vibrations during image capture will compromise the quality of the images.

2. Make sure the Micro-I apparatus is in a horizontal position: the air bubble under the glass on top of the apparatus should be positioned in the black circle (check perpendicular to the apparatus). If not, level the Micro-I using some paper.

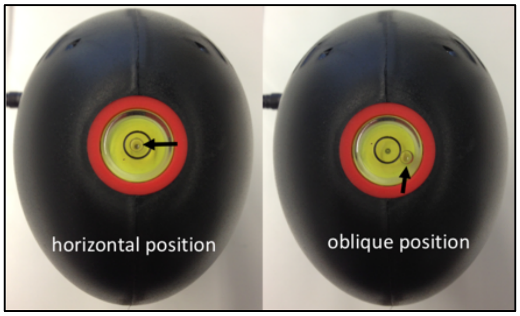

3. On the back, the Micro-I has two connections: one for the laptop connection (above) and one for the power supply (below).

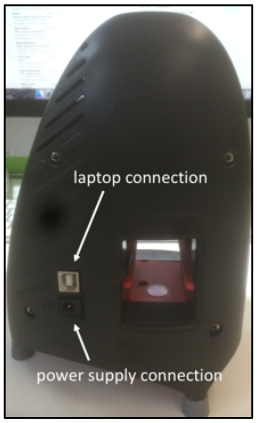

4. Connect the power supply cable to the Micro-I and to the power point (see Picture below).

5. Connect the Micro-I to the laptop: the USB end goes into the USB port at the right hand side of the laptop, the square end goes into the Micro-I.

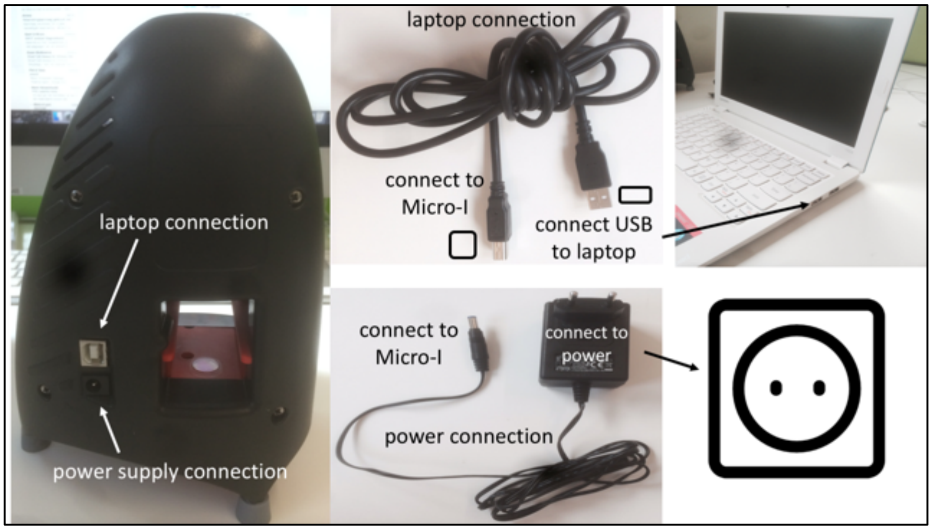

6. Initiate the FECPAK^{G2} Lab-Lite software by clicking on the desktop icon.

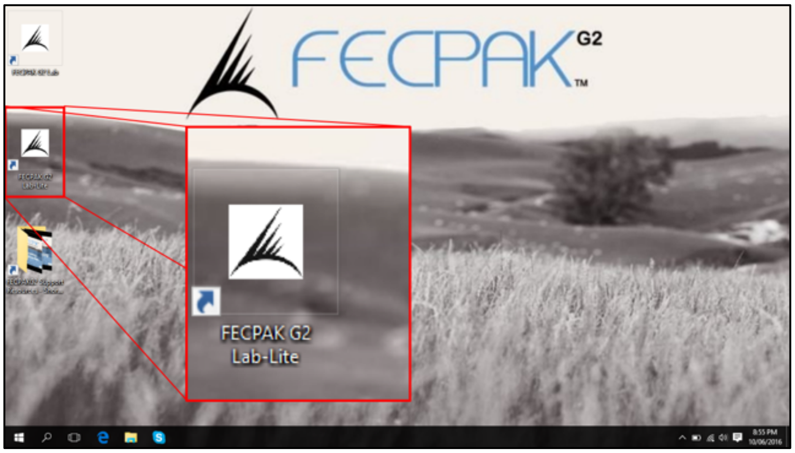

7. A login window opens: fill in user name and password, and click 'login'.

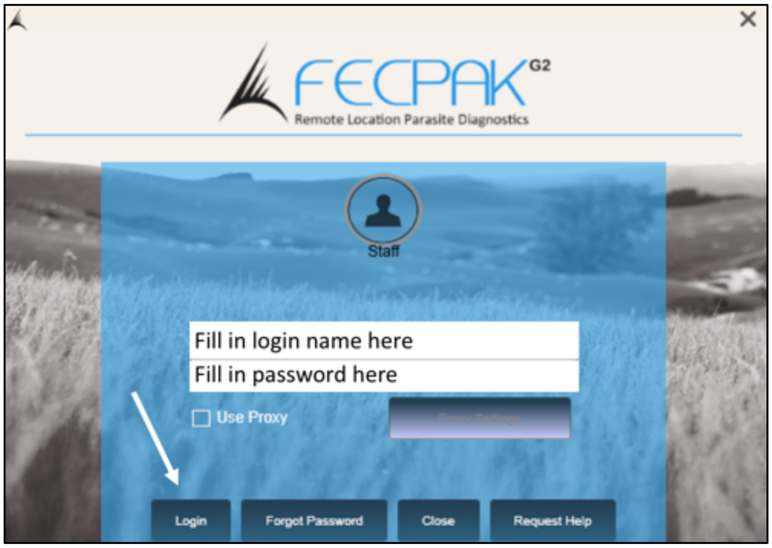

8. Go to the samples that have been submitted: click on 'View/Edit Test'.

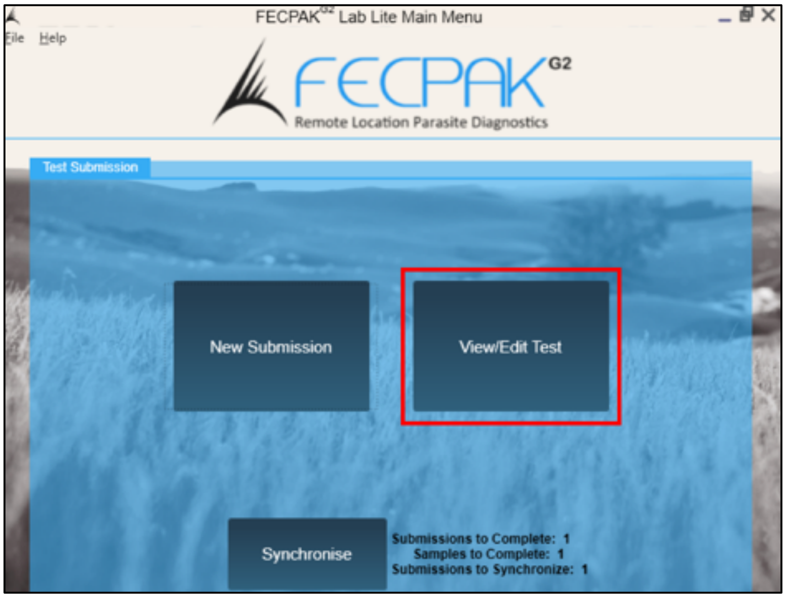

9. A window opens where you can see the different submissions. Select the submission and click on 'open' or double click on it.
<u>Note</u>: Submissions in red have are submission where no subjects have been entered. Submissions in blue are submissions where subjects have been entered, but these still require images. Submissions in green are submissions with subjects that have already images attached, but these have not yet been synchronized with the FECPAK^{G2} server, but are ready to be synchronized.

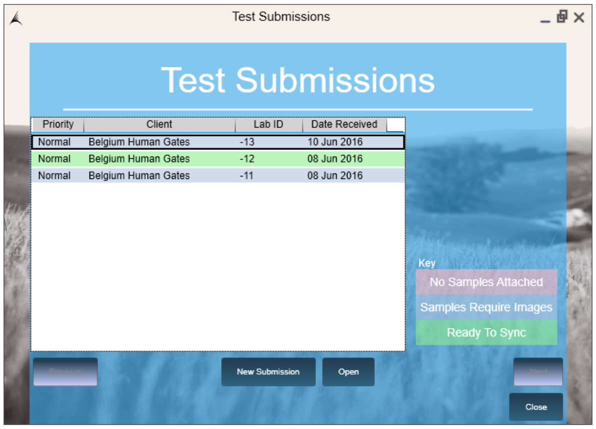

10. The list of samples appears. To open the sample for image capture: highlight sample and click ‘open sample’ or double click on the sample.

11. The window with data of the subject ID opens.

12. Click on ‘Add Images’.

13. A window ‘Capture Status’ opens.
    <u>Note:</u> The setting for ‘Sample Brightness’ is by default ‘Normal’. You can keep the default setting.

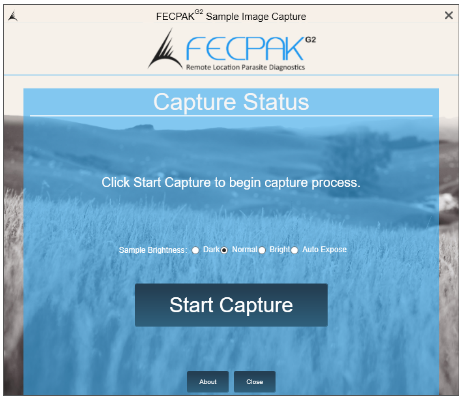

14. Remove the tape from the cassette, stick it on the Micro-I and place the cassette in the Micro-I. The pointed end of the cassette has to go in first. Gently push until you feel some resistance.

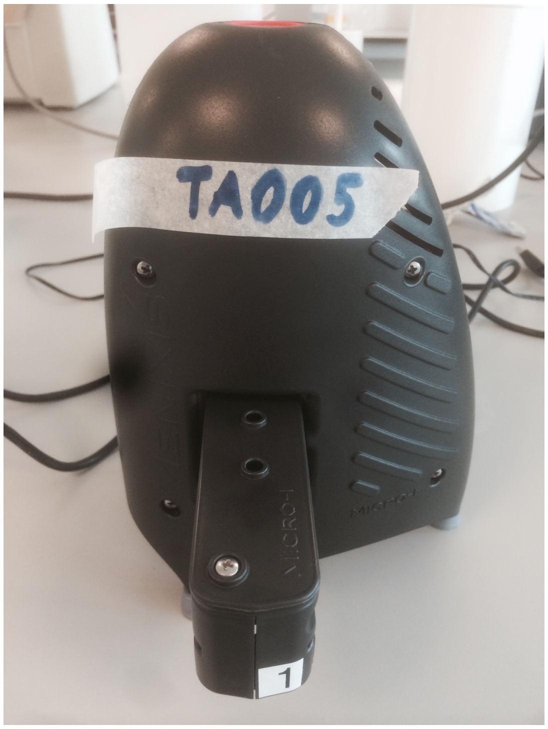

15. Click on start capture. The Micro-I will automatically read the both wells now. On the screen, you can see the remaining time needed to generate both images.

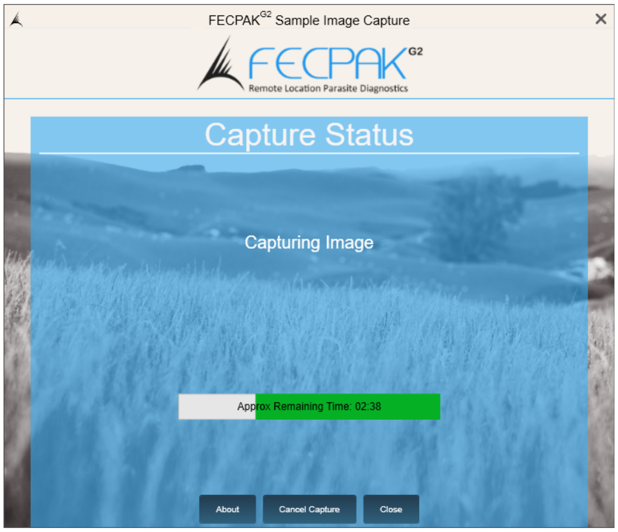

16. When the first picture is taken, it appears as an icon named 'well'.

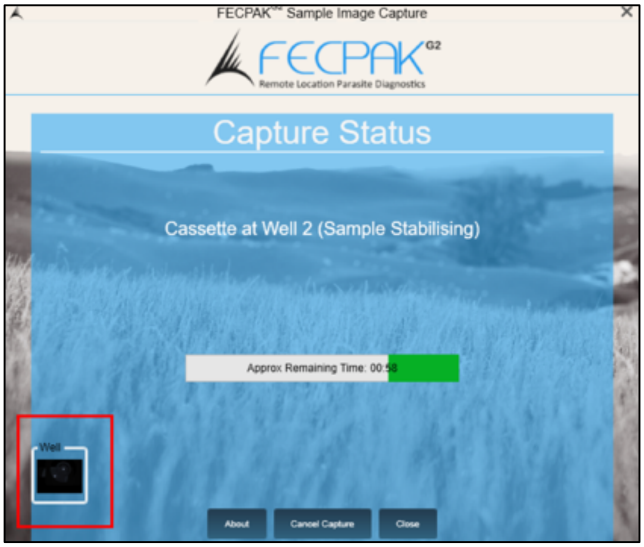

17. When both images are complete, you will see a notification 'Upload Complete', and you see 2 icons for the two images, both named 'Well'. The left icon is the first image, the right icon is the second image.

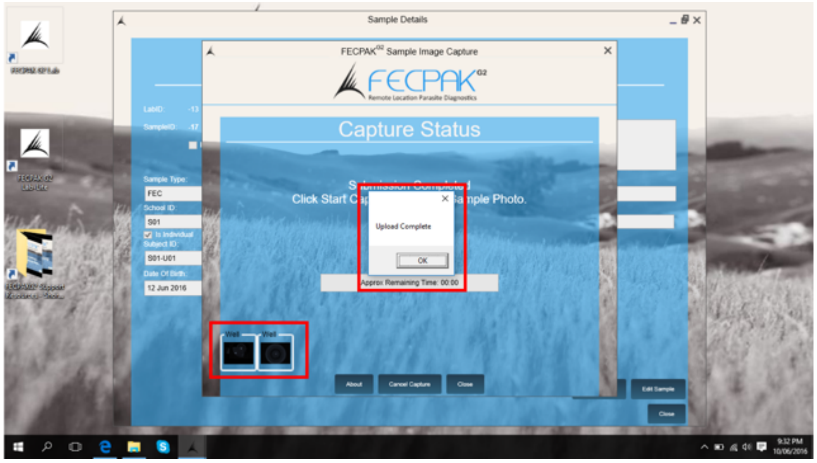

18. To check the quality of the two images, click on each icon and check the quality.

19. If quality is satisfactory (images appear sharp), take out the cassette from behind the Micro-I.

20. To take pictures of the next sample, click on 'close'. Do not click on 'Start Capture' as this will take pictures of the same subject ID. Click on the next 'subject ID' and take pictures as described above for the next sample.

3.5 Image mark-up

1. Open FECPAK^{G2} Lab Software on the desktop by clicking on the icon. The login window opens.

   <u>Note</u>: Mark-up of images can only be performed using the **FECPAK^{G2} Lab software**, <u>NOT</u> using the **FECPAK^{G2} Lab <u>Lite</u> software** (used to submit samples and to acquire images).

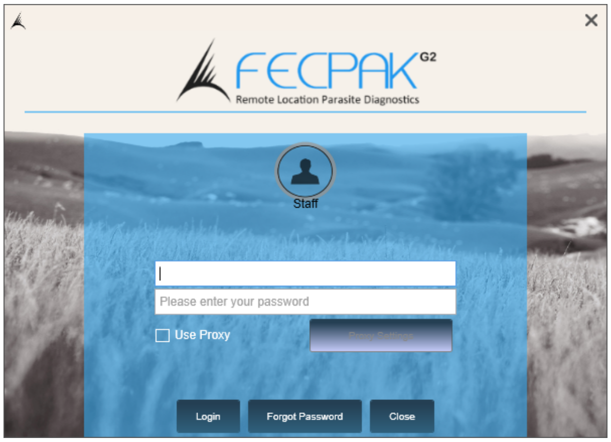

2. In the login window, fill in your login name in the first field and your password in the second field.

3. The Main Menu opens and three tabs are visible in the top left corner: Test Submission, Scan and Markup. Click on the Markup tab.

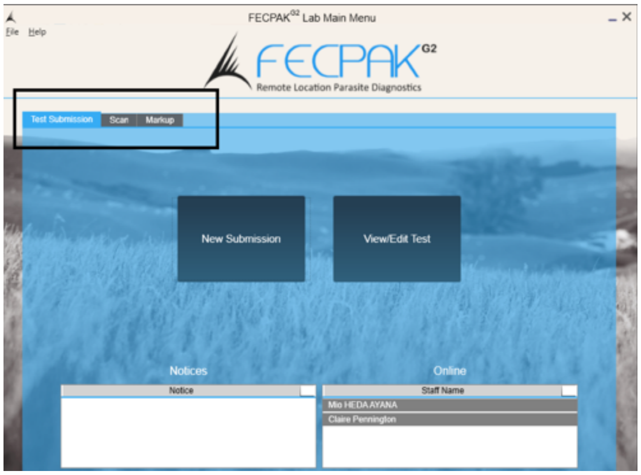

4. A window opens. In the 'Notices' field, you will see how many images need to be marked-up. In the example below, 3 samples are ready for mark-up.

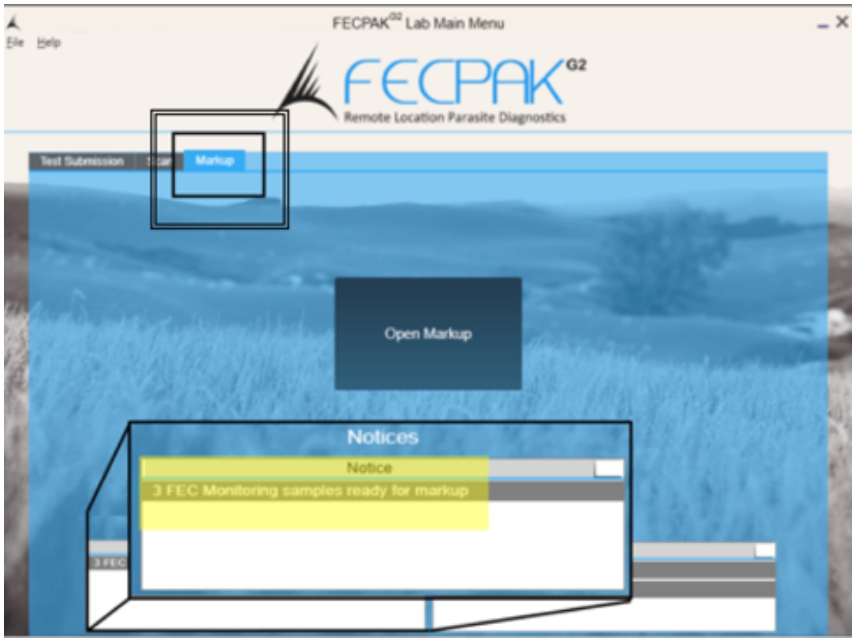

5. Click on the ‘Open Markup’ button.

6. Select the appropriate submission, the ‘Sample Markup’ window opens showing the TWO images of the selected subject.

   You can zoom in or out using the top slider. You can move the image using the left (vertical movement) and bottom (horizontal movement) sliders, or alternatively, using the left mouse bottom: click, hold in and move.

   Changing the appearance of the image can be done by changing the brightness, contrast, saturation and gamma sliders on the righthand side of the screen.

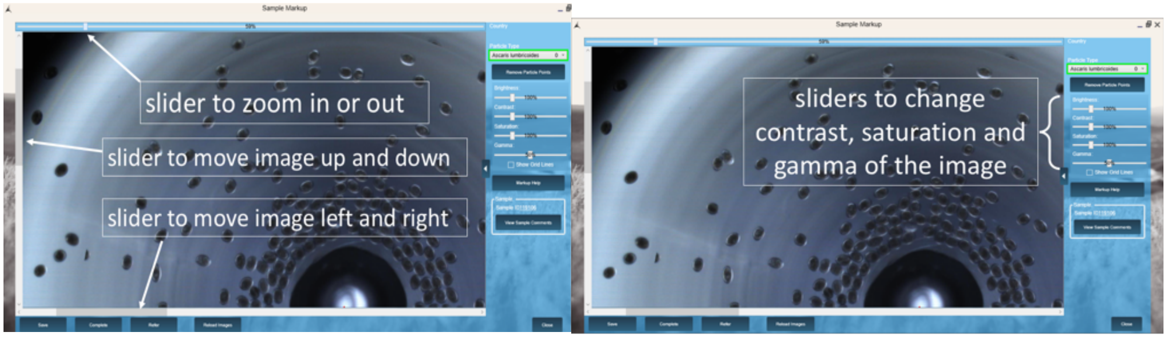

By ticking the ‘Show Grid Lines’ box, grid lines will appear. This might be handy in navigating or to get an idea on the scale of the image. By clicking on the button depicted

on the image below (right), an example of an *Ascaris*, *Trichuris* & hookworm egg is shown, with grids of the same scale as the grids that can be displayed in the image.

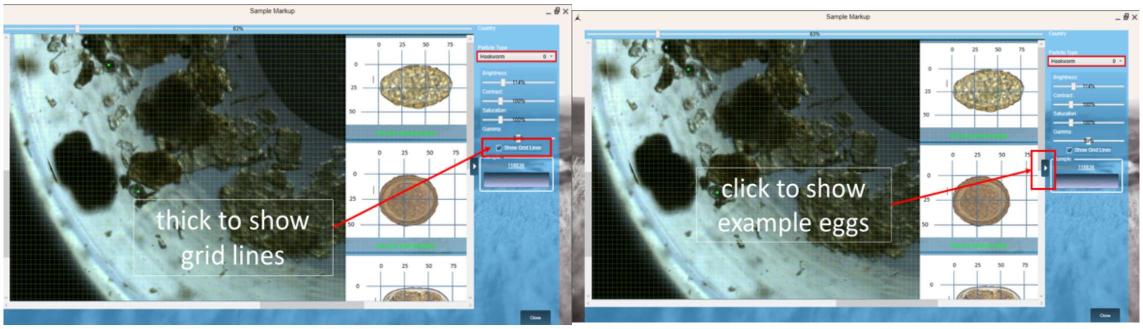

7. If the image settings (brightness, contrast, saturation and gamma) are satisfactory, you can start marking-up eggs. If not, adjust accordingly.

8. Choose a species from the dropdown ‘Particle Type’ in the top right corner. In the example below, ‘hookworm’ is selected. Screen both images systematically for the presence of hookworm eggs. If you see a hookworm egg, clicking on it will mark the egg with a dot. Repeat for *Ascaris* and *Trichuris*.

   Different species have different colored dots, as in the dropdown: *Ascaris* is green, hookworm is red and *Trichuris* is yellow. Alternatively, clicking the right mouse button when screening the image, will also give the dropdown in the image field, as depicted below.

   The total number of marked eggs for a species can be found in the dropdown next to the species names. In the example below, 3 *Ascaris* eggs have already been detected.

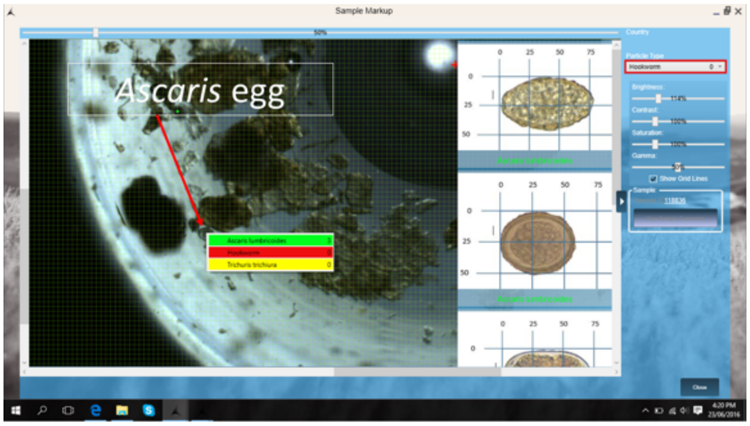

9. Click on ‘Complete’ to go to the next mark-up and repeat steps 6 – 8.

4. Cleaning and maintenance of the FECPAK^{G2} system

4.1. Cleaning of the cassettes

1. Pour out the suspension from the cassettes in the sink.

2. Rinse the cassettes with tap water.

   Caution: Avoid water at the bottom of the cassettes!

3. Gently dry the cassettes with tissue paper.

   Caution: Do not touch the glass rods.

4. Apply some drops of 0.5% Virkon to a cotton swab, and gently touch the glass rod of each well from a cassette with the cotton swab.

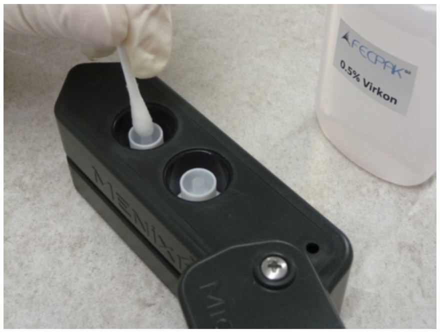

Note: Virkon will help improve the adhesion of the saline solution to the rod, hereby helping to maintain the quality of the pictures.

4.2. Cleaning of the sedimenters

1. Pour out the suspension from the sedimenters in the sink.

2. Rinse the sedimenters with tap water.

3. Dry the sedimenters using paper tissue.

4.3. Cleaning of the filters and cylinders

1. Open the cylinders and pour out the suspension from the cylinders in the sink.

2. Disassemble the filters from the filter top and place the filters and filter top in a beaker with water to soak.

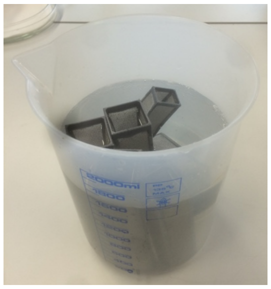

3. After soaking, rinse the filters, filter tops and cylinders thoroughly with tap water.

4. Dry the filters, filter tops and cylinders using paper tissue.

4.4. Cleaning of the tips

1. Put the tips in a beaker with water.

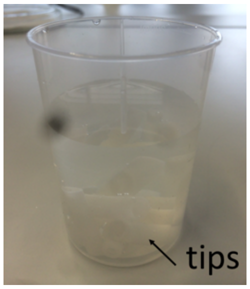

2. Rinse the tips using tap water and let the tips air-dry.
